# The jam session between muscle stem cells and the extracellular matrix in the tissue microenvironment

**DOI:** 10.1038/s41536-022-00204-z

**Published:** 2022-02-17

**Authors:** Mafalda Loreti, Alessandra Sacco

**Affiliations:** Development, Aging and Regeneration Program, Sanford Burnham Prebys Medical Discovery Institute, 10901N Torrey Pines Rd, La Jolla, CA 92037 USA

**Keywords:** Stem-cell niche, Regeneration

## Abstract

Skeletal muscle requires a highly orchestrated coordination between multiple cell types and their microenvironment to exert its function and to maintain its homeostasis and regenerative capacity. Over the past decades, significant advances, including lineage tracing and single-cell RNA sequencing, have contributed to identifying multiple muscle resident cell populations participating in muscle maintenance and repair. Among these populations, muscle stem cells (MuSC), also known as satellite cells, in response to stress or injury, are able to proliferate, fuse, and form new myofibers to repair the damaged tissue. These cells reside adjacent to the myofiber and are surrounded by a specific and complex microenvironment, the stem cell niche. Major components of the niche are extracellular matrix (ECM) proteins, able to instruct MuSC behavior. However, during aging and muscle-associated diseases, muscle progressively loses its regenerative ability, in part due to a dysregulation of ECM components. This review provides an overview of the composition and importance of the MuSC microenvironment. We discuss relevant ECM proteins and how their mutations or dysregulation impact young and aged muscle tissue or contribute to diseases. Recent discoveries have improved our knowledge about the ECM composition of skeletal muscle, which has helped to mimic the architecture of the stem cell niche and improved the regenerative capacity of MuSC. Further understanding about extrinsic signals from the microenvironment controlling MuSC function and innovative technologies are still required to develop new therapies to improve muscle repair.

## MuSC and their niche

The most abundant tissue in the human body is skeletal muscle, representing about 40% of total body weight. This tissue allows body posture, breathing, and voluntary movements, organism metabolism, and maintenance of body temperature^1^. Skeletal muscle is a highly organized tissue composed of blood vessels, nerves, connective tissue, and long multinucleated muscle cells named myofibers^2^. Myofibers harbor multiple proteins and enzymes, allowing the production of contractile forces for movements such as locomotion and posture^3^. Muscle stem cells (MuSC), also known as satellite cells, participate in postnatal myofiber growth^4^. However, when the tissue reaches homeostasis, their numbers decline as they enter and remain in a quiescent state of adult stages^5^. In 1961, Alexander Mauro identified satellite cells in a specialized niche between the myofiber sarcolemma and the basal lamina. He defined satellite cells as “merely dormant myoblasts… ready to recapitulate the embryonic development of skeletal muscle fiber when the main multinucleate cell is damaged”^6^.

**Fig. 1:**
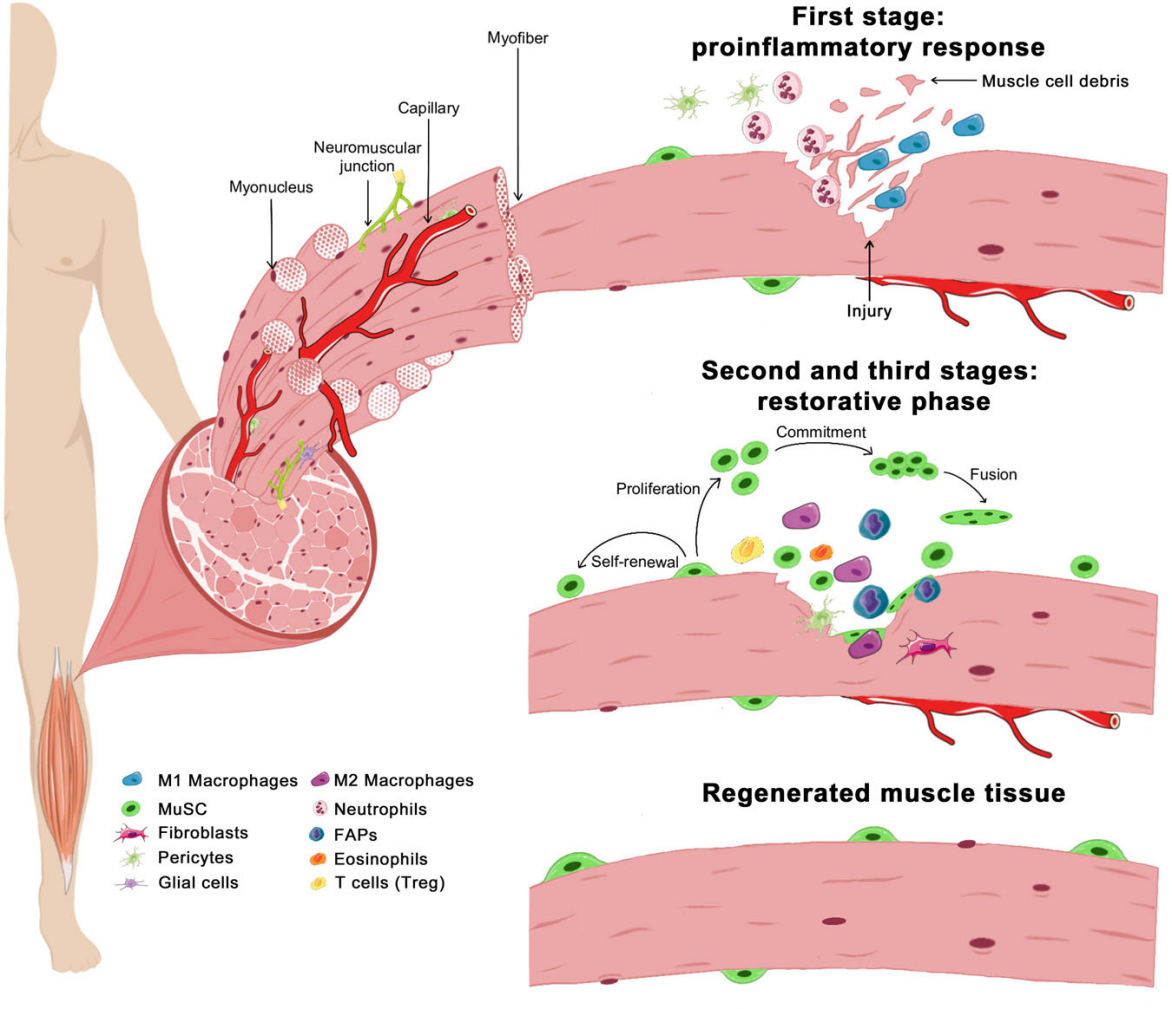
A schematic overview of different steps during muscle repair. Skeletal muscle tissue comprises multiple cell types and compartments, including multinucleated myofibers, blood vessels, and neuromuscular junctions. Upon injury, this tissue recruits several cell types (represented in the figure) to repair myofibers. The starting phase of muscle regeneration, also known as a proinflammatory response, is characterized by the infiltration of immune cells that will clear the damaged fibers from the injured site. During the first phase, neutrophils^224–228^ and proinflammatory macrophages (also known as M1) are required to clean the muscle cell debris and participate in the recruitment and activation of other cell types^229,230^. During the second stage, multiple cell types proliferate, including MuSC. Other immune cells, such as regulatory T cells (known as Treg)^231^ and eosinophils, also infiltrate the regenerating muscle after injury. Eosinophils stimulate FAPs expansion by forming a transitional niche favorable to clear necrotic debris and prevent FAPs differentiation into adipocytes^19^. In order to repair the damaged muscle, MuSC becomes activated, differentiate, and fuse to give rise to multinucleated myotubes. During this time, M1 macrophages are replaced by anti-inflammatory macrophages (also known as M2), which allows the restoration of the tissue. They express anti-inflammatory markers, several ECM-related genes, and growth factors (such as TGF-ß) and contribute to the remodeling of the MuSC niche. While M1 macrophages promote MuSC proliferation and prevent their premature differentiation, M2 macrophages boost MuSC commitment and formation of mature myotubes^61,232–234^. New fibers are thus formed and grow during the last phase, named the restorative phase. MuSC repopulates their stem cell pool at this stage, the injured site is remodeled, and the tissue is recovered and can return to homeostasis.

The niche is a local anatomic milieu instructing MuSC to participate in tissue formation, maintenance, and repair^7^. The conditional mouse model *Pax7*^*CreER*^*; R26R*^*DTA*^ allows the specific depletion of MuSC through the Cre-mediated activation (in PAX7-expressing cells) of diphtheria toxin fragment A (DTA), an inhibitor of protein translation that kills specifically DTA-positive cells. The use of this recombination-based lineage tracing mouse model showed that elimination of MuSC abrogates tissue repair upon acute injury^8–10^, thus suggesting that MuSC are the major source of muscle regeneration. Several intrinsic and extrinsic factors regulate MuSC function in the stem cell niche. Extrinsic signals sent by the niche are essential to maintain cell stemness, as described 40 years ago by Schofield for hematopoietic stem cells^11^. The stem cell niche plays a critical role in transmitting mechanical signals to MuSC to sense the structure, stiffness, and strength of the muscle^12^. The niche enables MuSC to maintain their quiescent state and provides them the ability to proliferate, migrate, self-renew, progress through the myogenic lineage, and differentiate to repair the damaged tissue (Fig. 1).

**Fig. 2:**
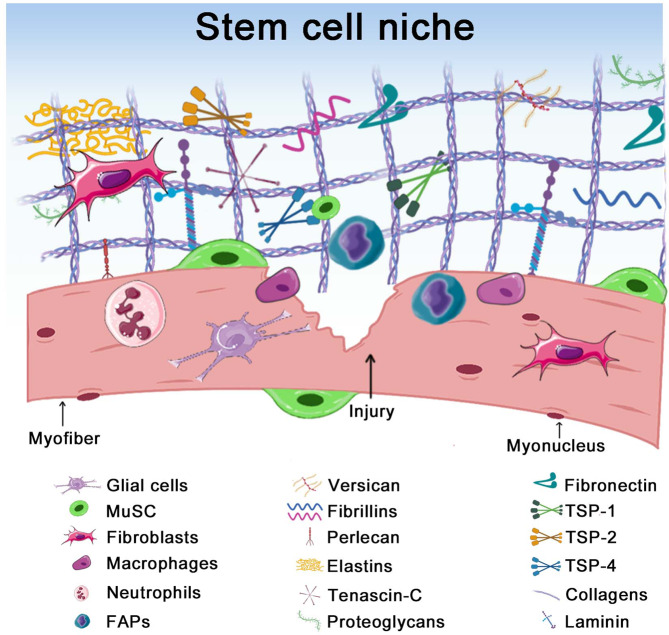
The stem cell niche in skeletal muscle and its ECM proteins. Upon tissue damage, there is a remodeling of the extracellular matrix. The niche is composed of different cell types that contribute to tissue repair and allow the expression of multiple ECM proteins. The most studied ones are represented in this figure.

**Fig. 3:**
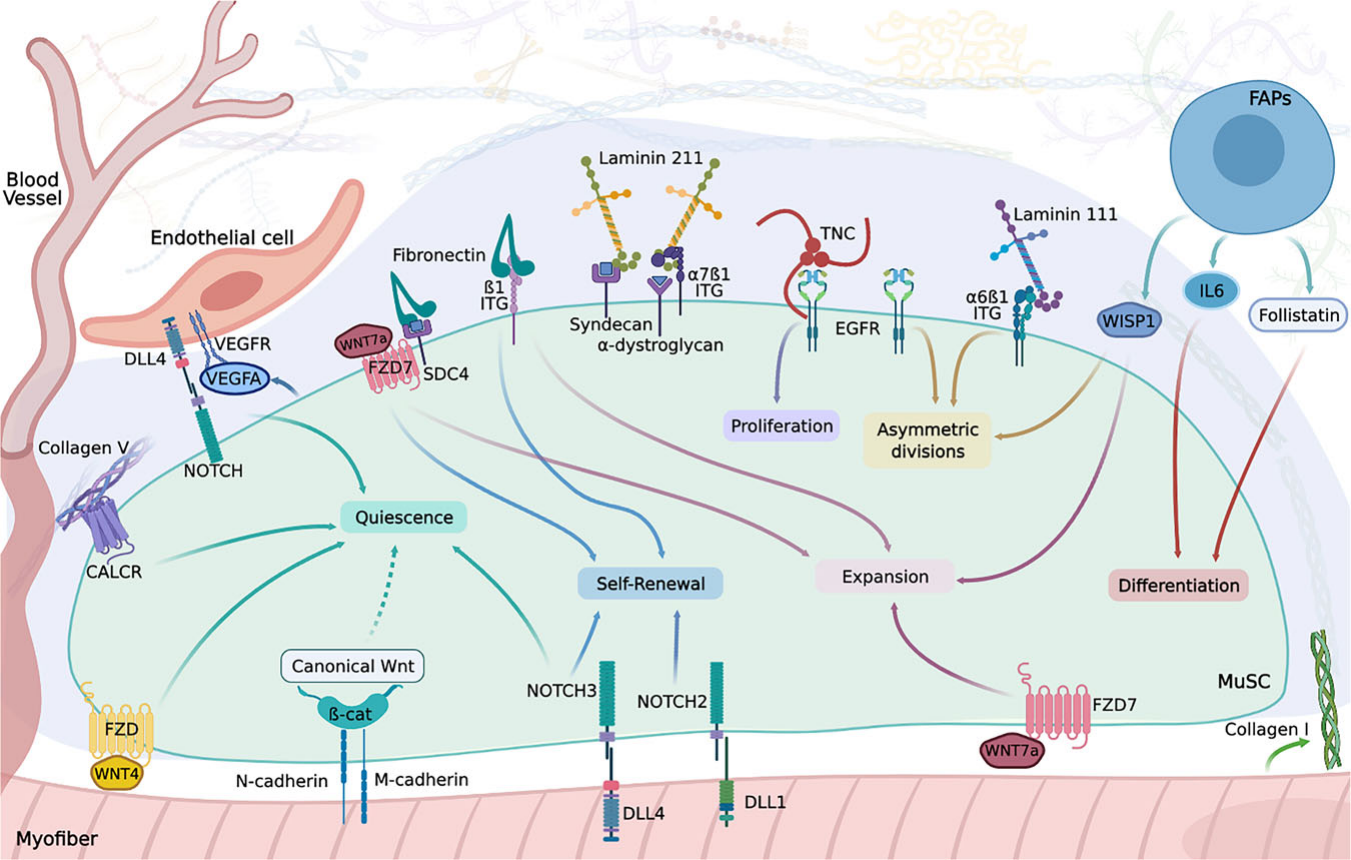
A schematic representation showing how MuSC interact with their microenvironment. MuSC are in direct contact with myofibers. These multinucleated cells secrete ECM proteins (as collagen-I) and soluble factors, including WNT4 and WNT7a, perceived through frizzled (FZD) receptors in the MuSC. As represented in this figure, MuSC directly interact with myofibers through Notch receptors and Dll ligands, and the cadherin-based adhesive junctions M- and N-cadherins. These adhesive junctions regulate MuSC quiescence through Canonical-Wnt (dotted arrow). However, ß-catenin activity has also been involved in the stimulation of MuSC differentiation^235^ (not shown in the figure). On the opposite side, MuSC are exposed to multiple ECM proteins in the basal lamina and their microenvironment. MuSC respond to ECM proteins through interaction with integrins (ITG), Calcitonin (CALCR), syndecans (including syndecan-4 (SDC4)), α-dystroglycan, and EGF receptors. These stem cells are able to interact with endothelial cells via Vegf and Notch signaling. MuSC fate is also regulated by signals emitted by FAPs (WISP1, IL6, and follistatin).

The ECM is a complex network within the stem cell niche (Fig. 2) that plays a critical role during tissue remodeling. Besides maintaining muscle structure and stiffness, the ECM also regulates the local concentration of soluble factors in the tissue^13^. Multiple muscle resident cells have been identified as major producers of these factors or contribute to ECM remodeling during tissue repair. For example, fibro-adipogenic progenitors (FAPs) are interstitial mesenchymal progenitors producing ECM components and soluble factors such as wnt1 inducible signaling pathway protein 1 (WISP1), bone morphogenic protein 1 (BMP1), and follistatin^14–18^, that support differentiation of myogenic progenitors, further aiding myogenesis^14,19,20^. Endothelial cells are another example of muscle-resident cells in the juxtavascular stem cell niche and are known to regulate MuSC quiescence and self-renewal through the Dll4-Notch pathway^21^ (Fig. 3).

In healthy adult tissue, the specific location and orientation of MuSC set a constant interaction with the basal lamina on the basal side and the myofibers on their apical side. In addition, myofibers play an essential role in regulating MuSC fate^22^. However, how MuSC perceive signals from their surrounding environment has been an emerging topic in the last years. For example, M-cadherin and N-cadherin, which are components of adhesive junctions, are expressed at sites of direct contact between MuSC and myofibers, and regulate MuSC-quiescence through the canonical Wnt/ß-Catenin signaling^23^. Likewise, the non-canonical Wnt pathway regulates the homeostatic maintenance of the MuSC pool^24,25^. The Brack group showed that Wnt4, secreted by the myofibers, positively regulates MuSC quiescence by activating Rho-GTPase and repressing yes-associated protein (YAP)^24^. Wnt7a, another secreted factor from the WNT family, has been shown to mediate symmetric MuSC expansion through its binding to frizzled-7 (FZD7) and Syndecan-4 (SDC4)^25,26^, two cell-surface receptors expressed in quiescent cells^25,27,28^.

Activation of the Notch signaling pathway requires direct cell-cell binding between MuSC and myofibers^29^, which includes the Delta-Like ligands (Dll) expressed by myofibers and the Notch receptors 1, 2, and 3 (NOTCH1, NOTCH2, NOTCH3) expressed by MuSC^30–32^. This signaling pathway participates in the maintenance of MuSC in a quiescent state^33,34^ and prevents their progression into the cell cycle^30,32,35^. Other studies have also identified Notch as the mediator of MuSC self-renewal^31^ through direct regulation of Pax7^36^.

All these studies have contributed to our understanding of the complexity of the MuSC niche and how signals coming from different sources can regulate MuSC stemness and myogenic properties. The use of lineage tracing and single-cell RNA sequencing has allowed the construction of a cell atlas of adult skeletal muscle in healthy and regenerative conditions and has uncovered significant heterogeneity of the different cell types in this tissue^37–42^. However, the relative contribution of each cell population to the ECM composition is still actively under study. Thus, understanding the MuSC niche composition and defining the role of ECM components is relevant for developing therapeutic interventions to improve muscle repair and ameliorate muscle-associated diseases.

In this review, we discuss the ECM composition of the MuSC microenvironment and summarize the relevance of multiple ECM proteins on MuSC function during muscle repair. Finally, we describe the impact of the ECM proteins in the niche on regulating MuSC function in muscle-associated diseases and during aging and discuss the developing approaches currently under study to better characterize the ECM and prospectively improve muscle healing.

## The ECM in the MuSC microenvironment

The basal lamina is a thin layer of the basement membrane, composed mainly of the ECM proteins Laminin and Collagen. It can be described as a biophysical barrier that provides mechanical support to the tissue and protects the MuSC from the external signals coming from their microenvironment, thus preventing their activation and entry into the myogenic program^43^.

In adults, cells are constantly rebuilding and remodeling the ECM to maintain tissue homeostasis^44–48^. The ECM mechanical properties change as a muscle grows and influence its length and tension. Muscle stiffness increases throughout life, and that can be explained by the biochemical composition of the ECM and its rearrangement^49,50^. As age stiffness increases and MuSC function is progressively impaired, it is important to understand how ECM components are regulated since some have been known as contributors to muscle tissue stiffness. Previous studies have explored whether substrate elasticity regulates MuSC properties and muscle regeneration. Indeed, culturing MuSC on pliant hydrogel mimicking the elasticity of the in vivo native muscle enhanced MuSC survival and self-renewal, prevented their differentiation, and maintained their ability to repair muscle upon transplantation, compared to traditional culture on rigid plastic^51^. This is an example showing that soft substrates enhanced MuSC function and stemness. However, there is an optimal elasticity of the tissue since the proliferation of primary myoblasts is influenced by a specific stiffness of the gel substrate, not being too soft or rigid^52^. Likewise, reducing the collagen content in muscle (in the collagen VI null mouse model) increased the elasticity of the tissue, leading to a loss of MuSC self-renewal^53^.

These studies revealed the sensitivity of MuSC to the biophysical properties of skeletal muscle. Consequently, it will be relevant to identify more components of the MuSC microenvironment that regulate tissue stiffness to develop approaches mimicking their native niche.

In the following sections, we describe how ECM proteins regulate MuSC and which approaches have been developed to bioengineer substrates to study biomechanical signals in vitro and in vivo. Three-dimensional (3D) scaffolds mimicking the properties and mechanical integrity of the native tissue are essential to understand how biomechanical signals modulate and/or stimulate MuSC function and fate and to develop tissue engineering approaches to improve tissue repair.

## The complexity of the ECM in skeletal muscle

Previous studies on mammary gland development have proposed a model in which there is a “dynamic reciprocity” between the ECM and mammary cells^54^. In fact, there is a bidirectional interaction between cells and their microenvironment^55^: through cell surface receptors, ECM components directly interact with cells and regulate their fate, proliferation, differentiation, migration, and adhesion (Fig. 3).

In skeletal muscle, the remodeling of the ECM represents an essential step during tissue repair since it impacts MuSC behavior and function. Myofibers are surrounded by ECM composed of glycoproteins, proteoglycans, collagens, matrix metalloproteinases (MMP), and matricellular proteins^56^. These factors have been shown to contribute to the regulation of MuSC function, muscle growth, and repair^57^. Furthermore, there is constant cooperation between ECM proteins and secreted molecules. For example, growth factors contribute to the production of the ECM proteins, and ECM proteins can either present or sequester growth factors to the cells^58^.

Below, we detail three categories of ECM proteins known to modulate MuSC function during muscle repair—collagens, fibronectin, and laminins, which have been extensively studied in the muscle field. Other ECM proteins, including fibrillins, tenascin-c (TnC), versican, periostin, and thrombospondins (TSP), are summarized in Table 1 and discussed in the next section, as they have been mostly studied in the context of muscle-associated diseases. Other components of the stem cell niche, including soluble factors, proteoglycans, and MMPs, have been previously reviewed in depth^12,44,59–61^.

**Table 1:** The functional regulation of ECM proteins in skeletal muscle and muscle-associated diseases.

Gene	Major roles	References
Collagen-I	It is highly expressed in undifferentiated C2C12 myoblasts. Differentiation of C2C12 is inhibited when these cells are treated with collagen-type I.	Alexakis et al.^67^
	It supports the myogenic progression of C2C12 cells when used as a biomaterial in 3D culture. Collagen-type I treatment impairs murine MuSC elongation of differentiation into myotubes.	Prüller et al.^70^
Collagen-V	It participates in the maintenance of MuSC in its quiescent niche.	Baghdadi et al.^68^
Collagen-VI (Col6)	Essential for structural support, maintenance, and differentiation of muscle.	Cescon et al.^76^
	Mutations in COL6 lead to Bethlem myopathies and Ullrich congenital muscular dystrophies.	Lampe and Bushby^129^
	It is highly expressed by freshly isolated MuSC and regulates their self-renewal.	Urciuolo et al.^53^
	*Col6α1*^*−/−*^ mouse model recapitulates Bethlem myopathy phenotype.	Bonaldo et al.; Grumati et al; Irwin et al.^130–132^
Fibronectin	Knockout murine adult models not viable — embryonic death at day 8.5.	George et al.^94^
	In skeletal muscle, it is transiently expressed during tissue remodeling. It promotes symmetric expansion and self-renewal of MuSC.	Bentzinger et al.^26^
	It is a preferential adhesion substrate for MuSC.	Lukjanenko et al.^98^
Laminin-211 (Merosin)	Laminin-211-deficient mice (*dy*^*w*^, mouse model for MDC1A)—a decrease of PAX7^+^ cells and increase of asymmetric cell divisions leading to failure of MuSC expansion; myofibers are smaller and muscle size is not recovered postnatally.	Nunes et al.^116^
Laminin-111	Laminin 111 can compensate for the loss of laminin-211 when used as a treatment in muscle tissue of *dy*^*w*^ mice.	Rooney et al.; Van Ry et al.^113,114^
	Laminin-111 treatment decreases muscle pathology in murine and canine models of Duchenne muscular dystrophy.	Barraza-Flores et al.; Goudenege et al.; Rooney et al.^142–144^
	Maintains MuSC polarity and mediates asymmetric cell division.	Rayagiri et al.^111^
Fibrillin-2 (Fbn2)	Mutations in the *FBN2* gene cause congenital contractural arachnodactyly (CCA) or Beals syndrome.	Belleh et al.; Lee et al.; Tsipouras et al.^204–206^
	Stimulates cell attachment of human dermal fibroblast.	Brinckmann et al.^207^
	Enhances lung epithelial regeneration by modulating basal epithelial stem cells proliferation, migration, and reducing cellular senescence.	Gilpin et al.^208^
	Absence of FBN2—reduced muscle mass, delay in forelimb muscle differentiation, contractures, infiltration of fat into the limb connective tissue space, and abnormal activation of BMP signaling.	Sengle et al.^172^
Fibrillin-1 (Fbn1)	Mutations in FBN1 cause Marfan syndrome.	Dietz et al.^179^
	Sequesters latent TGF-ß in the extracellular space.	Neptune et al.^180^
	Fbn1-deficient mice—delayed muscle regeneration, inhibited MuSC proliferation and differentiation, and increased TGF-ß activity.	Cohn et al.^181^
Periostin (Postn)	It is upregulated in dystrophic muscle and its deletion improves muscular dystrophy pathology.	Lorts et al.^145^
Tenascin-C (TnC)	Promotes MuSC expansion and enhances muscle repair.	Tierney et al.^69^
	It is secreted by necroptotic myofibers and promotes the proliferation of MuSC through EFGR interaction.	Zhou et al.^155^
	Modulates cell proliferation, survival, adhesion, migration, and differentiation of different cell types, including neural crest cells, fibroblasts, and tumor cells.	Akbareian et al.; Midwood et al.; Orend et al.; Saupe et al.; Tucker^209–213^
Thrombospondin-1 (TSP-1)	Accumulated in the skeletal muscle of patients with Dysferlinopathies (myopathy caused by a mutation in the dysferlin gene). Upregulation of TSP-1 correlates with higher chemotactic capacity and macrophage infiltration and activity into the muscle.	De Luna et al.; Urao et al.^165,166^
	Causes an increase in mitochondrial density in skeletal muscle, leading to a performance benefit and increased metabolic efficiency.	Frazier et al.^214^
	TSP-1 is an antiangiogenic regulator in skeletal muscle. Through the CD36 receptor, TSP-1 regulates basal skeletal muscle capillarity.	Audet et al.^215^
	TSP-1, expressed by visceral adipose tissues, induces muscle fibrosis, insulin resistance, and adipose tissue dysfunction during obesity progression. Besides, in cultured C2C12 myoblast cells, secreted TSP-1 inhibits insulin signaling associated with the activation of stress signaling (JNK, p38, and IKK).	Inoue et al.; Matsugi et al.^216,217^
Thrombospondin-2(TSP-2)	TSP-2 is transiently expressed after injury (hindlimb ischemia), including in muscle fibers.	Krady et al.^218^
Thrombospondin-4 (TSP-4)	Maintains structure and function of myotendinous junctions and regulates muscle integrity. Ameliorates vesicular trafficking of dystrophin protein. Mice lacking *TSP-4* showed a decreased muscle mass and poorer performance of limbs. Lack of TSP-4 caused spontaneous dystrophic changes in muscle with aging and accelerated muscular dystrophy in murine models.	Stenina-Adognravi and Plow; Vanhoutte et al.; Frolova et al.^169,219,220^
Versican (Vcan)	Regulates proliferation and inhibits differentiation of satellite cells isolated from pectoralis major muscle of F and RBC2 turkey lines. Clearance of VCAN by ADAMTS proteinases enhances the fusion of C2C12 myoblasts.	Stupka et al.; Velleman et al.^221,222^

In previous publications, several ECM proteins have been identified in the microenvironment of MuSC^69,98,223^. This table summarizes the major roles of some of those ECM proteins, with a particular focus on skeletal muscle and muscle-associated diseases.

### Collagens

Collagen is one of the most abundant ECM glycoproteins in the body and has a widespread distribution among tissues^56^. In vertebrates, there are 40 collagen genes identified that form 29 collagen-type homo- and/or heterotrimeric members^62^. Collagen is composed of three chains that wind together to form a triple-helical region responsible for the structural conformation of the protein^63^. The distribution of specific collagen heterotrimers is heterogeneous and can be tissue and region-specific. For instance, collagen IV (α3, α4, α5) and (α5)_2_(α6), is exclusive to neuromuscular junctions, whereas the same type of Collagen, but with different chains, is more abundant near the basement membrane of the myofibers^64,65^.

Collagen undergoes two major post-translational modifications in the endoplasmic reticulum: hydroxylation, which participates in its thermal and mechanical stability in the triple helix, and glycosylation, which regulates the collagen-assembled form^62,66^. Fibroblasts are one of the major producers of collagens. Other cell types such as chondrocytes, MuSC, and committed myoblasts can also secret several types of collagens, including type I, V, or VI^53,62,67–69^.

#### Type I Collagen

Type I collagen has been studied in vitro using the myoblast cell line C2C12 (immortalized mouse myoblast cell line) and MuSC associated with single isolated myofibers^67^. The Bou-Gharios group showed that Collagen-I is highly expressed in undifferentiated C2C12 myoblasts, and this expression is downregulated at the beginning of differentiation. Consistently, differentiation of C2C12 cells is inhibited when these cells are treated with soluble collagen-I^67^. However, more recently, it has been shown that when collagen type-I is used as a biomaterial in a 3D culture, it supports the myogenic progression of C2C12 myoblasts allowing their elongation, proliferation, attachment, and their alignment for myogenic differentiation and fusion into multinucleated myotubes^70^. The myogenic potential of immortalized human C25Cl48 myoblasts was also tested in 3D collagen type-I culture, but it was reduced, and these cells were unable to contract spontaneously^70^. Furthermore, isolated MuSC from mouse extensor digitorum longus muscle, cultured on Matrigel and embedded in collagen type-I for several days, could not elongate or form myotubes^70^. These findings reveal some of the challenges of studying ECM components in vitro and highlight the need to understand further their impact on MuSC in 2D versus 3D cultures, to better recapitulate the tissue microenvironment. Additional studies will be essential to determine how Collagen-I regulates MuSC differentiation and explore how this ECM protein contributes to skeletal muscle regeneration in vivo. Crystal structure analysis and in vitro studies using recombinant domains have detected collagen-I binding sites on integrins α1ß1 and α2ß1^71,72^ and showed an interaction with decorin (a small dermatan sulfate proteoglycan present in the ECM)^73^. To give an example, in corneal cells, ß1-integrin interacts with collagen-I to regulate cell survival^74^. However, it is still unclear whether a similar interaction occurs in skeletal muscle and whether it impacts MuSC function. Furthermore, the identification of pathways regulated by collagen-I required for the modulation of MuSC fate is indispensable to improve our understanding of its role in native tissues, as well as for the development of 3D cultures that better mimic the tissue microenvironment and can serve as useful ex vivo tissue modeling platforms.

#### Collagen V

Collagen-V (COLV) is a glycoprotein produced by MuSC in murine models^68^. In vitro, treatment with COLV delayed the entry of freshly isolated MuSC into the cell cycle, proliferation, and differentiation, while treatment with other collagens (COL-I and COL-VI) did not have this effect. Accordingly, specific deletion of collagen-V encoding genes in MuSC resulted in a spontaneous exit from quiescence and promoted differentiation, leading to the exhaustion of the MuSC pool^68^. Collagen-V is a ligand for the calcitonin receptor, a marker of quiescent MuSC, and this binding mediates the interaction with MuSC. In this study, the authors further showed that MuSC are retained in their niche, in a quiescent state, through Notch-Collagen-V-Calcitonin receptor signaling^68^. Further studies have shown that Notch signaling induces the transcription of the quiescence-specific microRNA-708 (miR708), which consequently inhibits tensin3, a component of the focal adhesion complex^75^. Through the Notch-miR708-Tensin3 axis, MuSC migration is inhibited, and quiescence is stabilized, thus maintaining the MuSC pool^75^. As Notch signaling is required to maintain MuSC quiescence and allows cell–cell communication, it will be relevant to broaden our understanding of ECM-interaction and Notch receptor/ligands on controlling MuSC function during muscle regeneration.

#### Collagen-VI

Collagen-VI (COL6) is one of the major components of the ECM and is ubiquitously expressed in multiple tissues, including skeletal muscle, nervous system, adipose tissue, skin, and cartilage^76^. It exerts several functions such as regulation of cell differentiation, inhibition of apoptosis, and maintenance of cell stemness. Until a few years ago, three chains, α1, α2, and α3, were known to be encoded by COL6A1, COL6A2, and COL6A3 genes. In 2008, three other chains were identified in mice and humans: α4, α5, and α6^77^. COL6 leads to the formation of the characteristic beaded microfilaments network in the basement membrane of muscle. This cell-adherent ECM protein is required for structural support, maintenance, and differentiation of muscle^77^. Collagen-VI has been described as being a vital component of the MuSC niche^53^. This ECM protein is adjacent to the surface of MuSC and highly expressed by freshly isolated MuSC while following MuSC activation, the expression of collagen-VI decreases. When *Col6α*^*−/−*^ murine muscles are subjected to injury, muscle regeneration is delayed and there is a significant reduction in MuSC numbers, suggesting that collagen-VI regulates MuSC self-renewal and is needed for the maintenance of the MuSC pool. In vitro experiments showed that collagen-VI promotes the maintenance and survival of PAX7-expressing MuSC. The authors demonstrated that collagen-VI is involved in regulating muscle stiffness and MuSC function depends on this mechanical property of the tissue. Transplantation of fibroblasts from wild-type mice into *Col6α*^*−/−*^ tibialis anterior muscles induced the production and deposition of collagen-VI and led to an increase in MuSC in collagen-VI deficient mice^53^. These studies define a relevant role of collagen-VI in the maintenance of the MuSC pool in their own niche. In vitro studies have shown that collagen-VI can bind multiple integrins^74,76,78,79^, including α1ß1 and α2ß1 in melanoma cells^78^. However, how collagen-VI communicates with MuSC to regulate their pool is still incompletely understood.

Overall, collagens are among the most abundant proteins in the body and regulate MuSC during muscle repair. Although the precise contribution of type-I collagen on regulating MuSC differentiation is still unclear, types-V and VI are needed to modulate self-renewal and maintain the stem cell pool. Additional studies are required to identify the cell surface receptors allowing the interaction between collagens and MuSC and other muscle-resident cell types to understand how they regulate MuSC function. Collagens can either directly bind to a MuSC-surface receptor or regulate the expression of soluble factors that will further modulate MuSC function. Similarly, these studies might contribute to a better understanding of how MuSC respond to the microenvironment stiffness. These discoveries are necessary to develop innovative tissue modeling approaches, including the engineering functional skeletal muscle in vitro.

### Fibronectin

Fibronectins are one of the major components of the ECM, mainly expressed during embryonic development and transiently upregulated during tissue remodeling^26,80–82^. Fibronectin is a multidomain glycoprotein with a high molecular weight, existing as a dimer composed of two identical subunits (monomers) (~250 kDa per subunit) linked covalently through C-terminal pair of disulfide bonds^83^. Each monomer is composed of 12 FN type I (FNI), 2 FN type II (FNII), and 15-17 FN type III (FNIII) repeats, which enable high-affinity binding to growth factors, including insulin-like growth factor binding-protein-3, fibroblast growth factor (FGF)-2, and vascular endothelial growth factor (VEGF)-A^84^. In humans, the gene encoding for fibronectin protein can generate up to 20 variants due to alternative mRNA splicing. This process is dependent on age, stage of development, and cell type, and each variant plays specific roles, including fibronectin secretion, adhesion, liver fibrosis, and skin wound healing^81^. In addition to alternative splicing, fibronectin can also undergo multiple post-translational modifications such as phosphorylation and glycosylation^85^. In mouse fibroblasts, phosphorylation of fibronectin participates in cell spreading, proliferation, and cell traction forces^86^. In human U2OS (human bone osteosarcoma) and HeLa (human cervical adenocarcinoma epithelial) cell lines, five N-glycosylation sites (N430, N528, N542, N1007, and N1244) were identified in the fibronectin protein. When N-linked glycans were cleaved using N-glycosidase F (PNGase F), adhesion and migration functions of fibronectin were significantly reduced, indicating that N-glycans of fibronectin are involved in cell adhesion and migration^87^. However, the post-translational modifications of fibronectin have not been extensively explored, either during muscle development or adult muscle tissue repair. In the future, it will be relevant to explore and compare whether fibronectin post-translational modifications are conserved and/or differ in these two contexts and whether they have a role in regulating MuSC function.

Fibronectin is present in both the ECM and in the plasma, thus existing as two distinct forms—insoluble and soluble, respectively. Fibronectin in the plasma is synthesized by hepatocytes in the liver, while in muscle, Fibronectin is produced by multiple cell types, including fibroblasts, chondrocytes, endothelial cells, myoblasts, myotubes, and MuSC^26,88,89^.

In multiple animal models, the deletion of fibronectin affects tissue development. For example, in zebrafish, the knockdown of fibronectin impairs somite formation and disrupts muscle morphogenesis^90^. In *Xenopus laevis*, fibronectin is essential for the attachment and migration of mesodermal cells. In addition, knockdown of fibronectin delays gastrulation and impairs convergence and extension, thus affecting body axis formation^82,91^. Furthermore, fibronectin is upregulated after appendage amputation in amphibians and is involved in multiple cellular mechanisms required for wound healing, such as cell growth and migration^92,93^. Finally, in murine models, embryos lacking this protein die before birth at day 8.5 and show deformed hearts, cardiovascular defects, and impaired formation of notochord and somites^94^.

In murine regenerating skeletal muscle, fibronectin is highly expressed in the microenvironment surrounding MuSC^26^. The Rudnicki group showed that fibronectin is expressed by MuSC during muscle repair and is strongly upregulated when MuSC are in an activated state and primed for myogenic commitment. This study demonstrated that committed muscle myogenic cells release high amounts of fibronectin into their niche. This work also showed that this ECM protein stimulates WNT7a activity through the frizzled-7 and syndecan-4 coreceptor complex to induce MuSC symmetric expansion and self-renewal^26^(Fig. 3). This ability of fibronectin in promoting MuSC self-renewal seems to be specific to adulthood since acute downregulation of fibronectin in fetal MuSC does not affect their expansion^69^. Furthermore, knockdown of fibronectin in adult MuSC leads to a decrease in the number of committed myogenic cells and a reduction in symmetric divisions numbers, therefore impairing muscle repair^26^. It has been previously shown that fibronectin delays myoblasts fusion in vitro, suggesting that this glycoprotein inhibits myogenic differentiation^95^. These findings, combined with Bentzinger’s results showing that once MuSC enter the differentiation program, fibronectin expression is downregulated^26^, suggest that low levels of Fibronectin are required to allow MuSC differentiation and promote tissue repair.

Besides syndecan-4, MuSC can also adhere to fibronectin through integrins (including alpha-5 (α5)-integrin)^96–98^). A recent study explored the interaction between fibronectin and integrins during nuclear positioning^96^. Multinucleated myofibers of skeletal muscle have a specific location for nuclei. Indeed, after fusion, nuclei migrate from the center to the periphery and stay underneath the plasma membrane^99^. The Gomes group has shown that myofibroblasts secrete fibronectin and deposit it at the surface of muscle cells to attract myonuclei to the cell periphery. Fibronectin secreted by myofibroblasts mediates peripheral nuclear positioning and directly modulates myofiber differentiation through activation of α5-integrin via focal adhesion kinases (FAK), SRC, and small Rho guanosine triphosphatase (GTPases) Cdc42^96^. Besides its function of regulating the migration of mesodermal cells during embryonic development and controlling the symmetric expansion and self-renewal of MuSC in adulthood, this work has shown that fibronectin is also required for nuclear movements within the developing myofibers.

In addition, a 3D time-lapse analysis exploring MuSC motility on single living fibers has demonstrated that primary cells plated on fibronectin showed a round shape and minimally adhered to this substrate. Also, the specific blockade of integrin-α5, a fibronectin-receptor, increased the velocity of MuSC, suggesting that fibronectin regulates MuSC motility^97^. Thus, it will be relevant to determine how fibronectin contributes to MuSC movements, for instance, to define the intracellular pathways that are activated once fibronectin binds to this integrin receptor. Also, it is currently unknown whether the deletion of fibronectin affects MuSC adhesion to the myofibers or to their stem cell niche. Nonetheless, it is already known that fibronectin promotes cell adhesion and ECM assembly^100^. However, this process is deregulated in aged MuSC, thus affecting the aged stem cell niche and the regenerative response of skeletal muscle^98^.

β1-integrin is a relevant MuSC receptor that contributes to the maintenance of MuSC homeostasis and supports MuSC expansion and self-renewal during muscle regeneration^100^. This group has shown that β1-integrin acts in cooperation with FGF2, a growth factor for MuSC^101^. Furthermore, it has been shown that fibronectin treatment on muscle cells affects FGF-ERK MAP kinases cascades^98^. Rozo et al. have tested FGF2 responsiveness in the presence of fibronectin in myoblasts lacking β1-integrin and have observed that this response was compromised, suggesting that there is a crosstalk between β1-integrin, fibronectin, and FGF2. This effect seems to be exerted through the binding of the arginine-glycine-aspartic acid motif^100^, a common integrin-binding domain present in multiple ECM proteins, including fibronectin and laminin^102^. Additional studies are necessary to characterize the specificity of this fibronectin-FGF2-β1-integrin interaction on regulating MuSC homeostasis.

### Laminins

Laminins are ubiquitously distributed throughout multiple tissues and laminin isoforms are tissue-specific. These ECM proteins play several biological roles, including tissue structure and maintenance, cell migration, adhesion, proliferation, differentiation, and survival^103^. Laminins are the major cell adhesive components of the basement membrane. They are heterotrimeric glycoproteins with three polypeptide chains α, ß, and γ encoded by different genes^56,104^. These chains bind together at the coiled-coil region of laminin, forming heterotrimeric isoforms with about ~400–900 kDa of molecular weight^89^. Mice lacking α or γ chains of laminin showed developmental defects leading to embryonic lethality or severe disorders after birth^105–107^, suggesting that these ECM proteins are essential for early development.

About 13–30% of the total molecular weight of these heavy glycoproteins is attributed to N-linked glycosylation^108,109^. In addition, N-glycosylation affects cell spreading, adhesion, scattering, and migration in human cells, as well as tumor growth and metastasis in animal models^109,110^, making this post-translational modification a potential target for the development of therapeutic approaches. However, these post-translational modifications have not been defined in skeletal muscle yet.

The function of laminins is dependent on ECM-binding proteins (e.g., collagen-IV), as well as cell-surface binding receptors, including α7ß1-integrin^103^ and α6ß1-integrin^111^. Additionally, laminins bind to the α-dystroglycan receptor, a component of the dystrophin–glycoprotein complex known to lead to muscular dystrophies when mutated^103^. These binding events are responsible for basement membrane assembly and mechanical linkage of the cell to this membrane^112^.

#### Laminin-111

The laminin-111 (chains α1ß1γ1) isoform is mainly expressed in embryonic skeletal muscle. In adults, activated MuSC secrete laminin-111 and this ECM protein is deposited into their basal lamina. It next binds its receptor α6ß1-integrin, leading to the maintenance of MuSC polarity and mediating asymmetric cell division^111^. This ECM protein has been studied in the context of muscular dystrophies, where it has been shown its potential treatment to alleviate these diseases^113,114^.

#### Laminin-211

Laminin-211 (chains α2ß1γ1), also named merosin, is the most abundant isoform expressed in the basement membrane and surrounds muscle fibers in adult skeletal muscle^103,115^. Laminin-211 controls muscle function through its binding to cell-surface receptors α7ß1-integrin, α-dystroglycan, and syndecans^103^. The consequences of the truncated deficiency form of the laminin α-2 chain (lacking the N-terminal LN domain) in skeletal muscle have been previously explored in murine tissues^116^. In this mouse model, myofibers are smaller and muscle size is not recovered postnatally. This phenotype is accompanied by a decrease in PAX7-positive MuSC and myogenin-positive cells, suggesting that these mice fail to generate sufficient numbers of MuSC to support fetal myofiber growth. Moreover, Nunes et al. observed an abnormal overactivation of JAK-STAT (Janus kinase-signal transducer and activator of transcription) signaling^116^. We and others have previously shown that STAT3 promotes MuSC progression into committed myogenic progenitors^57,117^. Also, stat3-knockdown stimulated symmetric MuSC divisions on cultured myofibers^118^. Thus, Nunes et al. suggested that in mice lacking laminin-211, activation of this signaling leads to an increase of the frequency of asymmetric cell divisions at the expense of symmetric cell divisions, impairing the expansion of the MuSC pool^116^.

The potential use of laminins as therapeutic tools has been previously explored by culturing isolated MuSC on the laminin-E8 fragment (C-terminal half of the coiled-coil region of laminin protein, which maintains the integrin-binding activity)^119^. The authors have demonstrated that the use of these fragments allowed the establishment of an artificial niche favorable for MuSC expansion and maintained MuSC in an undifferentiated state through modulation of JNK and p38 signaling^119^, two pathways essential for MuSC function^120–123^. Furthermore, murine or human cells cultured on these fragments showed enhanced engraftment and regenerative capacity when transplanted into mouse muscle tissues^119^.

Overall, laminins are essential ECM components present in the stem cell niche and surround MuSC to regulate their function. Additional studies will provide a better understanding of the contribution of these ECM proteins to the regulation of MuSC function. For example, glycosylation is associated with cell-cell communication and the regulation of intracellular signals. Furthermore, abnormal protein glycosylation of dystroglycan is the primary cause of some congenital muscular dystrophies^124^. Although laminin is known as a glycoprotein, whether these post-translational modifications of laminins occur in skeletal muscle and whether they impact this tissue or even MuSC function is still poorly explored. Moreover, using these ECM as a potential therapeutic tool appears to be very promising to improve MuSC regenerative capacity. Further studies are required to establish whether delivery of laminins into muscle tissue could improve MuSC expansion, function, and maintenance of the stem cell pool, especially in the context of muscular dystrophies. Also, these treatments’ efficiency could be evaluated to determine whether it could exert a long-term beneficial effect on muscle tissues.

## Dysregulation of ECM proteins compromises tissue repair in muscle-associated pathological contexts

Several studies have demonstrated the relevance of the stem cell niche in coordinating cell-cell communication and controlling MuSC function. ECM components are important not only to provide structural integrity to the niche and regulate tissue stiffness but also to modulate MuSC adhesion and migration during muscle repair^2,59^. However, ECM components significantly change their expression in muscle diseases and during aging, compromising tissue homeostasis and regenerative response^125–127^.

### ECM proteins in muscle-associated diseases

Multiple mutations or dysfunctions of ECM components are related to muscle-associated diseases^56^. The most common is primary myopathies and, in particular, muscular dystrophies. Muscular dystrophies are a group of inherited diseases characterized by progressive weakness and muscle loss^128^.

#### Collagen

Type VI collagen is one of the most studied ECM proteins in muscle since mutations in collagen-VI are responsible for several forms of human muscle diseases, including Bethlem myopathies and Ullrich congenital muscular dystrophies^129^. A genetic mouse model lacking one of the collagen-VI chains, the α1 (*Col6α*^*−/−*^), recapitulated the Bethlem myopathy phenotype. These mice showed myofiber necrosis and phagocytosis, changes in fiber diameter, mitochondrial dysfunction, and spontaneous apoptosis leading to myofibers degeneration and defective autophagy^130–132^.

#### Laminins

Mutation of laminins are associated with several human diseases, including Junctional epidermolysis bullosa (group of disorders characterized by abnormal fragility of the skin) or Congenital muscular dystrophies (group of diseases affecting skeletal muscles)^133^. A mouse model for human merosin-deficient congenital muscular dystrophy type 1A (MDC1A), named *dyW*, has been used to study the consequences of truncated deficiency of laminin α-2 chain in skeletal muscle^116^. This work has shown that the onset of this dystrophy occurs prenatally. In MCD1A, laminin-111 delivery has been studied as a substitute for the loss of laminin-211 in adults. Indeed, in the *dy*^*W*^ mouse model, the laminin-111 treatment prevented muscle disease progression and promoted muscle repair. This treatment also induced an increase in myofiber size and number of regenerating myofibers, and expression levels of PAX7 and myogenin, suggesting a restoration of muscle regeneration^113,114^. Therefore, the authors proposed that laminin-111 could counteract the loss of laminin-211 and prevent muscle disease progression of patients with this muscular dystrophy.

Duchenne muscular dystrophy (DMD) is the most common childhood form of muscular dystrophy, affecting 1 in 3500 male births and inducing progressive muscle weakness. Several invertebrate and mammalian models have been utilized to study the consequences of this genetic degenerative neuromuscular disorder, including *Caenorhabditis elegans*, *Drosophila melanogaster*, zebrafish, rats, pigs, cats, dogs, and several mouse models^134^, being the mdx mouse model the most widely used^135^. This lethal disease is caused by mutations in the dystrophin gene, leading to the lack of a dystrophin protein^136–139^. In myofibers, dystrophin connects the cytoskeleton to the ECM through the sarcolemma. Dystrophin is expressed not only in differentiated myofibers but also in activated MuSC, in which this protein regulates MuSC polarity and asymmetric division^140^. Dystroglycan is a membrane component of the dystrophin-glycoprotein complex and establishes the transmembrane link between laminin-2 and dystrophin^141^, indicating that ECM proteins can interact and regulate dystrophin. In *mdx* mice and Golden retriever muscular dystrophy dog models, laminin-111 treatment decreased muscle pathology by reducing muscle fiber damage and fibrosis^142–144^. These findings indicate that ECM components, particularly laminin-111, can be used as therapeutic tools for muscle diseases.

Finally, several ECM proteins change their expression in muscular dystrophies, including periostin, tenascin-c, TSP-1, and TSP-4.

#### Periostin

Periostin protein has been shown to accumulate at damage sites in human skeletal muscle biopsies from a patient with DMD^145^. This secreted ECM component has been described as a cell adhesion molecule, based on its protein structure and its effect on inducing osteoblast attachment and spreading in vitro^146^. Dystrophic mice lacking periostin exhibited enhanced myofiber regeneration and improvement in skeletal muscle function and structure^145^. This observation suggests that lack of periostin protects from muscular dystrophies progression and that this secreted ECM protein could be used as a potential therapeutic target for these pathologies. In other tissues, periostin has been shown to interact with collagens, fibronectin, and tenascin-c^147–149^. However, whether these interactions modulate MuSC function has not been extensively studied.

#### Tenascin-C

Tenascin-c (TNC) is expressed during embryonic development and decreases rapidly after birth^150,151^. In adults, it is transiently expressed during tissue injury and regeneration, including skeletal muscle^69,152–154^. In dystrophic muscles from mice, dogs, and humans, the glycoprotein TNC has been detected in degenerating and regenerating areas, whereas, in healthy adult skeletal muscle, this protein was not detectable^154^. However, little is known about the function of tenascin-c in this pathology.

MuSC secrete TNC, and this ECM protein promotes MuSC expansion and enhances muscle repair^69^. Another study exploring necroptosis, a type of programmed cell death, proposed that necroptotic myofibers promote MuSC proliferation by activating the TNC-epidermal growth factor receptor (EGFR) kinase cascade^155^. Indeed, the authors demonstrated that TNC is released by EGFR to promote MuSC proliferation^155^. Other studies have shown that activation of Egfr signaling promoted the production of myogenic progenitors through stimulation of asymmetric divisions^156,157^. Further studies are required to address whether EGF influences the proliferation of isolated MuSC and how the microenvironment regulates this factor. A recent study has further shown that TNC is also expressed by muscle glial cells activated upon nerve injury^158^, in accordance with the role of TNC on neuro-muscular junction formation and stabilization^159–161^. These studies suggest that MuSC, glial cells, and myofibers are sources of TNC in skeletal muscle. However, little is known on the potential of other muscle-resident cells to express this protein in other physiological and disease conditions. Furthermore, the molecular mechanism through which TNC promotes MuSC expansion and whether it regulates their function (including migration, differentiation, self-renewal, or quiescence) in a regenerative environment is still unclear. Additionally, initial evidence of activated necroptosis in dystrophin-deficient mouse and human muscles^162,163^ shows how preventing this type of programmed cell death could be a potential therapeutic tool to improve muscular dystrophies. Thus, further studies exploring the function of TNC in dystrophic models would improve the development of therapeutic approaches for this muscle disease.

#### Thrombospondins

Proteins from the TSP family are found within interstitial areas in human skeletal muscle tissues^164^ and are linked to several muscular dystrophies. Studies using the skeletal muscle of patients with dysferlinopathies (autosomal recessive muscle disorders caused by mutations in the dysferlin gene) showed an increased expression of TSP-1^165,166^. This ECM protein is secreted in response to inflammation^167^ and some studies have demonstrated that TSP-1 is expressed by muscle fibers and macrophages in muscles with this specific myopathy^165^. In dysferlin-deficient tissues, increased expression of TSP-1 in muscle fibers correlates with higher chemotactic activity promoting macrophages infiltration, suggesting that TSP-1 might contribute to inflammation^165^. The ubiquitin-proteasome system is known to recognize and remove unfolded or misfolded proteins. Further studies have explored whether the use of ubiquitin–proteasome inhibitors can prevent the degradation of dysferlin in immortalized myoblasts with dysferlinopathies^168^. Although the use of these inhibitors did not seem effective in restoring sarcolemma repair of damaged myofibers from patients, a decrease in TSP-1 release from myotubes was observed. The authors suggested that targeting TSP-1 could be a potential therapeutic approach to reduce inflammation in patients with dysferlinopathies^168^.

TSP-4 is transiently expressed in skeletal muscle upon injury or disease, including in dystrophic muscles (from human patients with Becker, DMD, and limb-girdle muscular dystrophies (LGMD2F)), where it is detected around macrophages infiltration and necrotic regions^169,170^. Vanhoutte et al. have used two models for muscular dystrophies: the *mdx* mice to study DMD and *sgcd* (deletion of δ-sarcoglycan) as a model for LGMD2F. Dystrophic mice lacking *TSP4* showed a significant worsening of these pathologies, suggesting that this ECM component might have a protective role^169^. Indeed, overexpression of TSP-4 in murine myofibers protected mice against muscular dystrophy. Additionally, a similar effect was observed in fruit flies, where TSP-4 treatment has rescued the muscular dystrophy phenotype, thus confirming that TSP-4 is protective and alleviates the dystrophic disease. The authors further uncovered a molecular mechanism where TSP-4 aids vesicular trafficking and chaperones dystrophin–glycoprotein and integrin attachment to the membrane, strengthening the stability and integrity of the myofiber membrane^169^. Further studies could explore whether TSP-1 and TSP-4 interact with myofibers and if they have an impact on MuSC function.

#### Fibrillins

Other ECM components, including members of the fibrillin family, have been shown to be associated with muscle diseases. Mutations in the gene coding for fibrillin-2 (FBN2), an ECM glycoprotein, cause Congenital contractural arachnodactyly. This genetic disorder is also known as Beals syndrome and affects connective and musculoskeletal tissues^171^. Genetic ablation of *FBN2* in mice leads to decreased muscle mass, increased white fat, delay in forelimb muscle development, and abnormalities in the musculature^172^. FBN2 is an important ECM constituent of all connective tissues and maintains tissue homeostasis by sequestering TGF-ß and BMP in a latent state, thus inhibiting activation of this signaling pathway^173–175^. Of note, in skeletal muscle, TGF-ß inhibits MuSC differentiation, thus impairing their regenerative capacity, and it has been previously reported that systemic delivery of TGF-ß receptor kinase inhibitor improves muscle regeneration^176,177^. Currently, the role of fibrillin-2 in muscle repair and especially in regulating MuSC function has not been comprehensively explored.

Marfan syndrome is a genetic disorder very similar to Beals syndrome and is the most common heritable disorder of connective tissues^178^. Mutations in the fibrillin-1 gene cause this autosomal-dominant disease. Individuals affected by this disorder have a myopathy and are incapable of increasing muscle mass even with exercise^179^. This gene codes for fibrillin-1 (FBN1), an ECM glycoprotein. Similar to fibrillin-2, this protein plays a critical role in sequestering TGF-ß on a latent state^180^. FBN1-deficient mice showed fibrosis and delayed muscle regeneration^181^. Although this ECM protein contributes to muscle repair by modulating TGF-ß, additional studies are needed to determine whether FBN1 communicates directly with MuSC through binding receptors or regulating soluble factors in the microenvironment.

Altogether, these studies have shown the contribution of ECM in the regulation of MuSC function in multiple muscle-associated diseases. Therefore, understanding the molecular dynamics of the stem cell niche and identifying other relevant ECM components will represent an essential step in developing new therapies to treat these pathologies.

### The stem cell niche during aging

Aging is associated with a decline in muscle mass, mobility, and quality of life. Aged individuals are confronted with physical limitations in part caused by age-related impairment of muscle regenerative ability. The aged population is more susceptible to injury and the muscle repair process is impaired compared to younger adults^182^. These impairments are associated with multiple cellular and molecular defects that directly affect MuSC function. With aging, the tissue regenerative potential is reduced due to a decline of cellular quiescence and self-renewal, as well as changes in cell proliferation and activation^5,123,126,183–187^.

Senescence, a cellular state induced by dysregulation of intrinsic and external signals leading to the functional exhaustion of the cell, affects MuSC function^188–191^. In geriatric (extremely old) mice, MuSC switch from quiescent to pre-senescence, a state leading to full irreversible senescence in response to regenerative pressure caused by derepression of p16INK4a (cyclin-dependent kinase inhibitor 2A). This affects MuSC activation and expansion, resulting in a reduction of the stem cell pool and capacity to participate in muscle repair^187^. The Muñoz–Cánoves team further showed that basal autophagy is crucial to maintain the MuSC quiescent state. Indeed, basal autophagy of aged MuSC is impaired, leading to an accumulation of toxic cellular waste, and as a result, MuSC enter into senescence. Thus, restoring autophagy can improve the regenerative potential of aged MuSC^192^.

It has been previously demonstrated that there is an abnormal deposition of ECM components in aged tissues, leading to fibrosis and increased tissue stiffness^193^. Indeed, Lukjanenko et al. have shown that some ECM components, including laminin, biglycan, and testican-1, are expressed at high levels in old muscles compared to young ones in homeostatic conditions^98^. Conversely, after injury, some ECM proteins that are upregulated in young muscles, fail to do so in aged muscles. For example, lower levels of fibronectin were detected after injury in old tissues compared to young ones. This team has studied the consequences of conditional deletion of fibronectin in young mice and showed that these mice recapitulated a premature aging phenotype. Moreover, fibronectin is the preferred adhesion substrate for mouse and human muscle progenitors and modulates multiple signaling pathways^98^ including differentiation-inducer pathways ERK and p38 mitogen-activated protein kinase^5,120,123^. Interestingly, fibronectin treatment rescued the adhesion capacity of aged MuSC, rejuvenated aged MuSC function by promoting their proliferative and myogenic potential in vivo, and improved muscle regeneration^98^.

A recent study has shown that aging is associated with increased muscle stiffness and pathogenic ECM architecture, resulting in aberrant myogenic progression of MuSC and affecting MuSC behavior^194^. Stearns et al. demonstrated that aging directly affects the gene expression of resident fibroblasts for the secretion of ECM components through YAP/transcriptional coactivator with PDZ‐binding motif (TAZ) signaling, promoting fibrogenic conversion of MuSC, thus affecting MuSC fate^194^.

MuSC function is not only affected by ECM components and muscle stiffness but can also be indirectly disturbed by other cell types present in the niche. It has been recently reported that FAPs activity is impaired with aging^18^. As we have previously described, FAPs secrete WISP1 (a protein regulating expansion and asymmetric commitment of MuSC), and since those cells lose their functionality with aging, WISP1 expression is affected in aged muscle. The authors demonstrated that loss of FAPs function combined with decreased WISP1 expression resulted in impaired MuSC function and muscle regeneration. Furthermore, WISP1 treatment restored MuSC myogenic potential in aged mice^18^. This is another research study showing that modulation of the stem cell niche can improve age-associated failures and promote muscle repair.

Altogether, these studies demonstrate how dysregulation of ECM components in the aged microenvironment impacts MuSC function. In agreement with these studies, the exposure of old mice to the young microenvironment significantly improved their regenerative potential, suggesting that modulation of systemic factors in aged tissues could be reversed^195^. Besides fibronectin, there are many other ECM components to be explored and these future studies will represent significant progress in the field to counteract muscle-aging-related pathologies, including sarcopenia, which is a chronic condition characterized by the age-related loss of skeletal muscle mass, strength, and performance^196^. Components from the MuSC microenvironment are promising targets for developing therapeutic interventions to reduce the adverse effects of sarcopenia.

## Approaches to defining the ECM composition and using it as a tool to improve skeletal muscle maintenance

Skeletal muscle is an oriented tissue and this specific organization is required to regulate its mechanical properties and keep the spatial organization of ECM and residing cells^197^. Thus, natural scaffolds retaining the mechanical integrity and the 3D ECM architecture are needed to recapitulate native tissue properties. Multiple studies showed that tissue decellularization is a powerful tool to generate naturally derived ECM and improve functional tissue regeneration upon transplantation in vivo^197–200^. In fact, implanted decellularized muscles promoted host cell migration, allowed a 3D reorganization of the myofibers, and restored the MuSC pool, thus promoting functional tissue regeneration^200^. However, in several of these studies, the composition of decellularized matrix is unknown, limiting our understanding of the presence and concentration of each ECM component as well as molecular mechanisms underlying their effects. Immunofluorescence experiments on tissues can be performed to enrich our knowledge about the ECM composition, however, these analyses are candidate-based approaches assessing only two or three ECM proteins at a time and rely heavily on the availability of specific antibodies. The recent development of single-cell proteomics approaches^201^ will overcome these limitations and enable a systematic assessment of skeletal muscle tissue composition in an unbiased manner. Finally, standard tissue digestion approaches result in loss of anatomical spatial information regarding the location of proteins in the tissue, while a central aspect of studying tissue composition is to be able to build a spatial mapping of cells and proteins, in order to evaluate their distribution and proximity to specific structures and microenvironments. Applying advanced technologies such as Nanostring Spatial Digital Profiling or STARmap^202^ for 3D in situ RNA sequencing or multiplex protein detection would allow single-cell analyses and protein distribution in intact issues.

As an alternative approach, in vitro cultures can be used for studying ECM proteins. Unfortunately, it is challenging to investigate ECM proteins in this context due to the lack of spatial cues as well as other tissue components that may interact with ECM proteins and modify their function. While 3D cultures are more versatile than 2D ones and enable us to recapitulate several features of native tissues, they still suffer from our limited understanding of tissue composition. The use of biomaterials able to mimic tissue properties at both the structural and functional level has significantly advanced the development of artificial matrices^203^. These biomaterials have been utilized both in vitro and in vivo by injection into muscle tissues. In order to mimic the MuSC-microenvironment and improve muscle repair, multiple aspects need to be taken into account to design suitable scaffolds. The architecture of this biomaterial must mimic the stiffness of the tissue to provide the mechanical properties, and also, it has to allow cell growth, motility, adhesion, and function, including self-renewal and differentiation. Furthermore, this artificial tissue requires biocompatibility with the tissue to avoid any inflammatory response, and it has to be biodegradable to be progressively replaced by the newly formed tissue^203^.

Overall, the integration of these new technologies will allow us to dissect skeletal muscle tissue composition, perform direct comparisons in different physiological and pathological contexts, and identify new approaches to modulate tissue microenvironments to promote MuSC function and tissue repair.

## Conclusion and perspectives

Several studies have contributed to deepening our knowledge about the composition of the stem cell niche, including the discovery of new ECM proteins and their function in regulating MuSC and muscle repair. The construction of a cell atlas of muscle tissues is definitively an advance in the muscle field, which is contributing to the discovery of the producers of ECM components. Extending our knowledge on the cooperation between muscle-resident cell types and MuSC is essential to decipher the behavior of MuSC and the MuSC–microenvironment dynamics. This will provide a better understanding of how those muscle-resident cell types communicate with MuSC and contribute to regulating their fate and function, either with direct physical interaction or by secreting factors, including ECM proteins. These discoveries will demonstrate that not only biochemical signals but also the physical cell-cell interactions are an important factor in controlling MuSC function and behavior, as shown in previous studies.

In recent years, several studies have identified a critical role of the microenvironment in instructing MuSC behavior and temporally coordinating tissue repair and maintenance. Several ECM components have been demonstrated to modulate MuSC self-renewal and differentiation or to contribute to maintaining their quiescent state. Some of the ECM proteins we have described are transiently expressed during healing processes, suggesting that there is temporal coordination of ECM proteins to modulate MuSC. In fact, after an acute injury in healthy tissue, appropriate deposition of a transient matrix has a positive role, and it is necessary to repair the damage efficiently. However, chronic inflammation leads to a persistent accumulation of ECM components, which can be explained by the excessive accumulation of their own producers, such as fibroblast-like cells, which frequently occur in aged and dystrophic tissues. Further studies are essential to explore the original source of ECM components and identify the signaling pathways associated with these factors, to understand whether they modulate MuSC function and how MuSC respond to these signals in homeostatic *versus* regenerative conditions. Although several studies have contributed to deepening our knowledge about the stem cell niche, future additional studies exploring the composition of the niche and manipulating ECM components in vivo are necessary to improve the regenerative potential of MuSC and alleviate muscle-associated diseases.

## References

[1] Kim KM, Jang HC, Lim S (2016). Differences among skeletal muscle mass indices derived from height-, weight-, and body mass index-adjusted models in assessing sarcopenia. Korean J. Intern. Med..

[2] Dinulovic I, Furrer R, Handschin C (2017). Plasticity of the muscle stem cell microenvironment. Adv. Exp. Med. Biol..

[3] Sambasivan R, Tajbakhsh S (2015). Adult skeletal muscle stem cells. Results Probl. Cell Differ..

[4] Buckingham M (2003). The formation of skeletal muscle: from somite to limb. J. Anat..

[5] Chakkalakal JV, Jones KM, Basson MA, Brack AS (2012). The aged niche disrupts muscle stem cell quiescence. Nature.

[6] Mauro A (1961). Satellite cell of skeletal muscle fibers. J. Biophys. Biochem. Cytol..

[7] Yin H, Price F, Rudnicki MA (2013). Satellite cells and the muscle stem cell niche. Physiol. Rev..

[8] Murphy MM, Lawson JA, Mathew SJ, Hutcheson DA, Kardon G (2011). Satellite cells, connective tissue fibroblasts and their interactions are crucial for muscle regeneration. Development.

[9] Lepper C, Partridge TA, Fan C-M (2011). An absolute requirement for Pax7-positive satellite cells in acute injury-induced skeletal muscle regeneration. Development.

[10] Sambasivan R (2011). Pax7-expressing satellite cells are indispensable for adult skeletal muscle regeneration. Dev. Camb. Engl..

[11] Schofield R (1978). The relationship between the spleen colony-forming cell and the haemopoietic stem cell. Blood Cells.

[12] Thomas K, Engler AJ, Meyer GA (2015). Extracellular matrix regulation in the muscle satellite cell niche. Connect. Tissue Res..

[13] Gattazzo F, Urciuolo A, Bonaldo P (2014). Extracellular matrix: a dynamic microenvironment for stem cell niche. Biochim. Biophys. Acta.

[14] Joe AWB (2010). Muscle injury activates resident fibro/adipogenic progenitors that facilitate myogenesis. Nat. Cell Biol..

[15] Mozzetta C (2013). Fibroadipogenic progenitors mediate the ability of HDAC inhibitors to promote regeneration in dystrophic muscles of young, but not old Mdx mice. EMBO Mol. Med..

[16] Armand A-S (2003). Expression and neural control of follistatin versus myostatin genes during regeneration of mouse soleus. Dev. Dyn..

[17] Juban G (2018). AMPK activation regulates LTBP4-dependent TGF-β1 secretion by pro-inflammatory macrophages and controls fibrosis in duchenne muscular dystrophy. Cell Rep..

[18] Lukjanenko L (2019). Aging disrupts muscle stem cell function by impairing matricellular WISP1 secretion from fibro-adipogenic progenitors. Cell Stem Cell.

[19] Heredia JE (2013). Type 2 innate signals stimulate fibro/adipogenic progenitors to facilitate muscle regeneration. Cell.

[20] Lemos DR (2015). Nilotinib reduces muscle fibrosis in chronic muscle injury by promoting TNF-mediated apoptosis of fibro/adipogenic progenitors. Nat. Med..

[21] Verma M (2018). Muscle satellite cell cross-talk with a vascular niche maintains quiescence via VEGF and notch signaling. Cell Stem Cell.

[22] Bischoff R (1990). Interaction between satellite cells and skeletal muscle fibers. Dev. Camb. Engl..

[23] Goel AJ, Rieder M-K, Arnold H-H, Radice GL, Krauss RS (2017). Niche cadherins control the quiescence-to-activation transition in muscle stem cells. Cell Rep..

[24] Eliazer S (2019). Wnt4 from the Niche controls the mechano-properties and quiescent state of muscle stem cells. Cell Stem Cell.

[25] Le Grand F, Jones AE, Seale V, Scimè A, Rudnicki MA (2009). Wnt7a activates the planar cell polarity pathway to drive the symmetric expansion of satellite stem cells. Cell Stem Cell.

[26] Bentzinger CF (2013). Fibronectin regulates Wnt7a signaling and satellite cell expansion. Cell Stem Cell.

[27] Cornelison DD, Filla MS, Stanley HM, Rapraeger AC, Olwin BB (2001). Syndecan-3 and syndecan-4 specifically mark skeletal muscle satellite cells and are implicated in satellite cell maintenance and muscle regeneration. Dev. Biol..

[28] Tanaka KK (2009). Syndecan-4 expressing muscle progenitor cells in the SP engraft as satellite cells during muscle regeneration. Cell Stem Cell.

[29] Lake RJ, Tsai P-F, Choi I, Won K-J, Fan H-Y (2014). RBPJ, the major transcriptional effector of notch signaling, remains associated with chromatin throughout mitosis, suggesting a role in mitotic bookmarking. PLOS Genet..

[30] Fujimaki S (2018). Notch1 and Notch2 coordinately regulate stem cell function in the quiescent and activated states of muscle satellite cells. Stem Cells.

[31] Yartseva V (2020). Heterogeneity of satellite cells implicates DELTA1/NOTCH2 signaling in self-renewal. Cell Rep..

[32] Low S, Barnes JL, Zammit PS, Beauchamp JR (2018). Delta-like 4 activates notch 3 to regulate self-renewal in skeletal muscle stem cells. Stem Cells.

[33] Bjornson CRR (2012). Notch signaling is necessary to maintain quiescence in adult muscle stem cells. Stem Cells.

[34] Mourikis P (2012). A critical requirement for notch signaling in maintenance of the quiescent skeletal muscle stem cell state. Stem Cells.

[35] Mourikis P, Gopalakrishnan S, Sambasivan R, Tajbakhsh S (2012). Cell-autonomous Notch activity maintains the temporal specification potential of skeletal muscle stem cells. Development.

[36] Wen Y (2012). Constitutive Notch activation upregulates Pax7 and promotes the self-renewal of skeletal muscle satellite cells. Mol. Cell. Biol..

[37] Dellavalle A (2011). Pericytes resident in postnatal skeletal muscle differentiate into muscle fibres and generate satellite cells. Nat. Commun..

[38] Giordani L (2019). High-dimensional single-cell cartography reveals novel skeletal muscle-resident cell populations. Mol. Cell.

[39] Liu N (2017). A Twist2-dependent progenitor cell contributes to adult skeletal muscle. Nat. Cell Biol..

[40] Malecova B (2018). Dynamics of cellular states of fibro-adipogenic progenitors during myogenesis and muscular dystrophy. Nat. Commun..

[41] Oprescu SN, Yue F, Kuang S (2020). Single-cell isolation from regenerating murine muscles for RNA-sequencing analysis. STAR Protoc..

[42] Petrany MJ (2020). Single-nucleus RNA-seq identifies transcriptional heterogeneity in multinucleated skeletal myofibers. Nat. Commun..

[43] Sanes JR (2003). The basement membrane/basal lamina of skeletal muscle. J. Biol. Chem..

[44] Forcina L, Miano C, Pelosi L, Musarò A (2019). An overview about the biology of skeletal muscle satellite cells. Curr. Genomics.

[45] Christov C (2007). Muscle satellite cells and endothelial cells: close neighbors and privileged partners. Mol. Biol. Cell.

[46] Naba A (2016). The extracellular matrix: tools and insights for the “omics” era. Matrix Biol..

[47] Rullman E (2009). Endurance exercise activates matrix metalloproteinases in human skeletal muscle. J. Appl. Physiol..

[48] Timmons JA (2005). Modulation of extracellular matrix genes reflects the magnitude of physiological adaptation to aerobic exercise training in humans. BMC Biol..

[49] Alnaqeeb MA, Al Zaid NS, Goldspink G (1984). Connective tissue changes and physical properties of developing and ageing skeletal muscle. J. Anat..

[50] Wood LK (2014). Intrinsic stiffness of extracellular matrix increases with age in skeletal muscles of mice. J. Appl. Physiol..

[51] Gilbert P (2010). Substrate elasticity regulates skeletal muscle stem cell self-renewal in culture. Science.

[52] Boonen KJM, Rosaria-Chak KY, Baaijens FPT, van der Schaft DWJ, Post MJ (2009). Essential environmental cues from the satellite cell niche: optimizing proliferation and differentiation. Am. J. Physiol..

[53] Urciuolo A (2013). Collagen VI regulates satellite cell self-renewal and muscle regeneration. Nat. Commun..

[54] Bissell MJ, Hall HG, Parry G (1982). How does the extracellular matrix direct gene expression?. J. Theor. Biol..

[55] Thorne, J. T. et al. Dynamic reciprocity between cells and their microenvironment in reproduction. *Biol. Reprod*. **92**, 25, 1-10 (2015). 10.1095/biolreprod.114.121368 PMC4434933 25411389

[56] Theocharis AD, Manou D, Karamanos NK (2019). The extracellular matrix as a multitasking player in disease. FEBS J..

[57] Tierney MT (2014). STAT3 signaling controls satellite cell expansion and skeletal muscle repair. Nat. Med..

[58] Schultz GS, Wysocki A (2009). Interactions between extracellular matrix and growth factors in wound healing. Wound Repair Regen..

[59] Gillies AR, Lieber RL (2011). Structure and function of the skeletal muscle extracellular matrix. Muscle Nerve.

[60] Evano B, Tajbakhsh S (2018). Skeletal muscle stem cells in comfort and stress. NPJ Regen. Med..

[61] Mashinchian O, Pisconti A, Le Moal E, Bentzinger CF (2018). The muscle stem cell niche in health and disease. Curr. Top. Dev. Biol..

[62] Sorushanova A (2019). The collagen suprafamily: from biosynthesis to advanced biomaterial development. Adv. Mater..

[63] Wu, M. & Crane, J. S. Biochemistry, Collagen Synthesis. in *StatPearls* (StatPearls Publishing, 2019). 29939531

[64] Legay C, Dobbertin A (2020). Collagens at the vertebrate neuromuscular junction, from structure to pathologies. Neurosci. Lett..

[65] Sanes JR, Engvall E, Butkowski R, Hunter DD (1990). Molecular heterogeneity of basal laminae: isoforms of laminin and collagen IV at the neuromuscular junction and elsewhere. J. Cell Biol..

[66] Kadler KE, Baldock C, Bella J, Boot-Handford RP (2007). Collagens at a glance. J. Cell Sci..

[67] Alexakis C, Partridge T, Bou-Gharios G (2007). Implication of the satellite cell in dystrophic muscle fibrosis: a self-perpetuating mechanism of collagen overproduction. Am. J. Physiol..

[68] Baghdadi MB (2018). Notch/CollagenV/CalcR reciprocal signalling retains muscle stem cells in their niche. Nature.

[69] Tierney MT (2016). Autonomous extracellular matrix remodeling controls a progressive adaptation in muscle stem cell regenerative capacity during development. Cell Rep..

[70] Prüller J, Mannhardt I, Eschenhagen T, Zammit PS, Figeac N (2018). Satellite cells delivered in their niche efficiently generate functional myotubes in three-dimensional cell culture. PLoS ONE.

[71] Xu Y (2000). Multiple binding sites in collagen type I for the integrins α1β1 and α2β1*. J. Biol. Chem..

[72] Boraschi-Diaz, I., Wang, J., Mort, J. S. & Komarova, S. V. Collagen type I as a ligand for receptor-mediated signaling. *Front. Phys*. **5**, 1–11 (2017).

[73] Schönherr E, Hausser H, Beavan L, Kresse H (1995). Decorin-type I collagen interaction: presence of separate core protein-binding domains *. J. Biol. Chem..

[74] Howell SJ, Doane KJ (1998). Type VI collagen increases cell survival and prevents anti-beta 1 integrin-mediated apoptosis. Exp. Cell Res..

[75] Baghdadi MB (2018). Notch-induced miR-708 antagonizes satellite cell migration and maintains quiescence. Cell Stem Cell.

[76] Cescon M, Gattazzo F, Chen P, Bonaldo P (2015). Collagen VI at a glance. J. Cell Sci..

[77] Fitzgerald J, Rich C, Zhou FH, Hansen U (2008). Three novel collagen VI chains, alpha4(VI), alpha5(VI), and alpha6(VI). J. Biol. Chem..

[78] Pfaff M (1993). Integrin and Arg-Gly-Asp dependence of cell adhesion to the native and unfolded triple helix of collagen type VI. Exp. Cell Res..

[79] Tulla M (2001). Selective binding of collagen subtypes by integrin alpha 1I, alpha 2I, and alpha 10I domains. J. Biol. Chem..

[80] de Almeida PG, Pinheiro GG, Nunes AM, Gonçalves AB, Thorsteinsdóttir S (2016). Fibronectin assembly during early embryo development: a versatile communication system between cells and tissues. Dev. Dyn..

[81] ffrench-Constant C (1995). Alternative splicing of fibronectin—many different proteins but few different functions. Exp. Cell Res..

[82] Darribère T, Schwarzbauer JE (2000). Fibronectin matrix composition and organization can regulate cell migration during amphibian development. Mech. Dev..

[83] Pankov R, Yamada KM (2002). Fibronectin at a glance. J. Cell Sci..

[84] Martino MM, Hubbell JA (2010). The 12th–14th type III repeats of fibronectin function as a highly promiscuous growth factor-binding domain. FASEB J..

[85] Paul JI, Hynes RO (1984). Multiple fibronectin subunits and their post-translational modifications. J. Biol. Chem..

[86] Yalak G, Shiu J-Y, Schoen I, Mitsi M, Vogel V (2019). Phosphorylated fibronectin enhances cell attachment and upregulates mechanical cell functions. PloS ONE.

[87] Hsiao C-T (2017). Fibronectin in cell adhesion and migration via N-glycosylation. Oncotarget.

[88] Hynes RO, Yamada KM (1982). Fibronectins: multifunctional modular glycoproteins. J. Cell Biol..

[89] Xu, J. & Mosher, D. Fibronectin and other adhesive glycoproteins. in *The Extracellular Matrix: an Overview* (ed. Mecham, R. P.) 41–75 (Springer Berlin Heidelberg, 2011). 10.1007/978-3-642-16555-9_2.

[90] Snow CJ, Peterson MT, Khalil A, Henry CA (2008). Muscle development is disrupted in zebrafish embryos deficient for fibronectin. Dev. Dyn..

[91] Davidson LA, Marsden M, Keller R, DeSimone DW (2006). Integrin α5β1 and fibronectin regulate polarized cell protrusions required for xenopus convergence and extension. Curr. Biol..

[92] Calve S, Odelberg SJ, Simon H-G (2010). A transitional extracellular matrix instructs cell behavior during muscle regeneration. Dev. Biol..

[93] Johnson MB (2017). Topical Fibronectin Improves Wound Healing of Irradiated Skin. Sci. Rep..

[94] George EL, Georges-Labouesse EN, Patel-King RS, Rayburn H, Hynes RO (1993). Defects in mesoderm, neural tube and vascular development in mouse embryos lacking fibronectin. Development.

[95] Podleski TR, Greenberg I, Schlessinger J, Yamada KM (1979). Fibronectin delays the fusion of L6 myoblasts. Exp. Cell Res..

[96] Roman W, Martins JP, Gomes ER (2018). Local arrangement of fibronectin by myofibroblasts governs peripheral nuclear positioning in muscle cells. Dev. Cell.

[97] Siegel AL, Atchison K, Fisher KE, Davis GE, Cornelison DDW (2009). 3D timelapse analysis of muscle satellite cell motility. Stem Cells.

[98] Lukjanenko L (2016). Loss of fibronectin from the aged stem cell niche affects the regenerative capacity of skeletal muscle in mice. Nat. Med..

[99] Cadot B, Gache V, Gomes ER (2015). Moving and positioning the nucleus in skeletal muscle—one step at a time. Nucleus.

[100] Rozo M, Li L, Fan C-M (2016). Targeting β1-integrin signaling enhances regeneration in aged and dystrophic muscle in mice. Nat. Med..

[101] Johnson SE, Allen RE (1995). Activation of skeletal muscle satellite cells and the role of fibroblast growth factor receptors. Exp. Cell Res..

[102] Bellis SL (2011). Advantages of RGD peptides for directing cell association with biomaterials. Biomaterials.

[103] Holmberg J, Durbeej M (2013). Laminin-211 in skeletal muscle function. Cell Adhes. Migr..

[104] Yurchenco PD, Wadsworth WG (2004). Assembly and tissue functions of early embryonic laminins and netrins. Curr. Opin. Cell Biol..

[105] Meng X (2003). Targeted inactivation of murine laminin gamma2-chain gene recapitulates human junctional epidermolysis bullosa. J. Invest. Dermatol..

[106] Ryan MC, Lee K, Miyashita Y, Carter WG (1999). Targeted disruption of the LAMA3 gene in mice reveals abnormalities in survival and late stage differentiation of epithelial cells. J. Cell Biol..

[107] Smyth N (1998). The targeted deletion of the LAMC1 gene. Ann. N. Y. Acad. Sci..

[108] Fujiwara S, Shinkai H, Deutzmann R, Paulsson M, Timpl R (1988). Structure and distribution of N-linked oligosaccharide chains on various domains of mouse tumour laminin. Biochem. J..

[109] Kariya Y (2008). N-Glycosylation of laminin-332 regulates its biological functions. J. Biol. Chem..

[110] Granovsky M (2000). Suppression of tumor growth and metastasis in Mgat5-deficient mice. Nat. Med..

[111] Rayagiri SS (2018). Basal lamina remodeling at the skeletal muscle stem cell niche mediates stem cell self-renewal. Nat. Commun..

[112] Yurchenco PD, McKee KK, Reinhard JR, Rüegg MA (2018). Laminin-deficient muscular dystrophy: molecular pathogenesis and structural repair strategies. Matrix Biol..

[113] Rooney JE, Knapp JR, Hodges BL, Wuebbles RD, Burkin DJ (2012). Laminin-111 protein therapy reduces muscle pathology and improves viability of a mouse model of merosin-deficient congenital muscular dystrophy. Am. J. Pathol..

[114] Van Ry,PM, Minogue P, Hodges BL, Burkin DJ (2014). Laminin-111 improves muscle repair in a mouse model of merosin-deficient congenital muscular dystrophy. Hum. Mol. Genet..

[115] Patton BL, Miner JH, Chiu AY, Sanes JR (1997). Distribution and function of laminins in the neuromuscular system of developing, adult, and mutant mice. J. Cell Biol..

[116] Nunes AM (2017). Impaired fetal muscle development and JAK-STAT activation mark disease onset and progression in a mouse model for merosin-deficient congenital muscular dystrophy. Hum. Mol. Genet..

[117] Sala D (2019). The Stat3-Fam3a axis promotes muscle stem cell myogenic lineage progression by inducing mitochondrial respiration. Nat. Commun..

[118] Price FD (2014). Inhibition of JAK-STAT signaling stimulates adult satellite cell function. Nat. Med..

[119] Ishii K (2018). Recapitulation of extracellular LAMININ environment maintains stemness of satellite cells in vitro. Stem Cell Rep..

[120] Bernet JD (2014). p38 MAPK signaling underlies a cell-autonomous loss of stem cell self-renewal in skeletal muscle of aged mice. Nat. Med..

[121] Charville GW (2015). Ex vivo expansion and in vivo self-renewal of human muscle stem cells. Stem Cell Rep..

[122] Troy A (2012). Coordination of satellite cell activation and self-renewal by par-complex-dependent asymmetric activation of p38α/β MAPK. Cell Stem Cell.

[123] Cosgrove BD (2014). Rejuvenation of the muscle stem cell population restores strength to injured aged muscles. Nat. Med..

[124] Endo T, Toda T (2003). Glycosylation in congenital muscular dystrophies. Biol. Pharm. Bull..

[125] Frangogiannis NG (2017). The extracellular matrix in myocardial injury, repair, and remodeling. J. Clin. Invest..

[126] Brack AS, Rando TA (2007). Intrinsic changes and extrinsic influences of myogenic stem cell function during aging. Stem Cell Rev..

[127] Cianflone E (2019). Adult cardiac stem cell aging: a reversible stochastic phenomenon?. Oxid. Med. Cell. Longev..

[128] Chang NC, Chevalier FP, Rudnicki MA (2016). Satellite cells in muscular dystrophy—lost in polarity. Trends Mol. Med..

[129] Lampe AK, Bushby KMD (2005). Collagen VI related muscle disorders. J. Med. Genet..

[130] Bonaldo P (1998). Collagen VI deficiency induces early onset myopathy in the mouse: an animal model for Bethlem myopathy. Hum. Mol. Genet..

[131] Grumati P (2010). Autophagy is defective in collagen VI muscular dystrophies, and its reactivation rescues myofiber degeneration. Nat. Med..

[132] Irwin WA (2003). Mitochondrial dysfunction and apoptosis in myopathic mice with collagen VI deficiency. Nat. Genet..

[133] Mcgowan KA, Marinkovich MP (2000). Laminins and human disease. Microsc. Res. Tech..

[134] McGreevy JW, Hakim CH, McIntosh MA, Duan D (2015). Animal models of Duchenne muscular dystrophy: from basic mechanisms to gene therapy. Dis. Model. Mech..

[135] Bulfield G, Siller WG, Wight PA, Moore KJ (1984). X chromosome-linked muscular dystrophy (mdx) in the mouse. Proc. Natl Acad. Sci. USA.

[136] Campbell KP, Kahl SD (1989). Association of dystrophin and an integral membrane glycoprotein. Nature.

[137] Ervasti JM, Ohlendieck K, Kahl SD, Gaver MG, Campbell KP (1990). Deficiency of a glycoprotein component of the dystrophin complex in dystrophic muscle. Nature.

[138] Hoffman EP, Brown RH, Kunkel LM (1987). Dystrophin: the protein product of the Duchenne muscular dystrophy locus. Cell.

[139] Mercuri E, Bönnemann CG, Muntoni F (2019). Muscular dystrophies. Lancet Lond. Engl..

[140] Dumont NA (2015). Dystrophin expression in muscle stem cells regulates their polarity and asymmetric division. Nat. Med..

[141] Ehmsen J, Poon E, Davies K (2002). The dystrophin-associated protein complex. J. Cell Sci..

[142] Barraza-Flores P (2019). Laminin-111 protein therapy enhances muscle regeneration and repair in the GRMD dog model of Duchenne muscular dystrophy. Hum. Mol. Genet..

[143] Goudenege S (2010). Laminin-111: a potential therapeutic agent for Duchenne muscular dystrophy. Mol. Ther. J. Am. Soc. Gene Ther..

[144] Rooney JE, Gurpur PB, Burkin DJ (2009). Laminin-111 protein therapy prevents muscle disease in the mdx mouse model for Duchenne muscular dystrophy. Proc. Natl Acad. Sci. USA.

[145] Lorts A, Schwanekamp JA, Baudino TA, McNally EM, Molkentin JD (2012). Deletion of periostin reduces muscular dystrophy and fibrosis in mice by modulating the transforming growth factor-β pathway. Proc. Natl Acad. Sci. USA.

[146] Horiuchi K (1999). Identification and characterization of a novel protein, periostin, with restricted expression to periosteum and periodontal ligament and increased expression by transforming growth factor beta. J. Bone Miner. Res..

[147] Kii I (2006). Periostin is an extracellular matrix protein required for eruption of incisors in mice. Biochem. Biophys. Res. Commun..

[148] Norris RA (2007). Periostin regulates collagen fibrillogenesis and the biomechanical properties of connective tissues. J. Cell. Biochem..

[149] Takayama G (2006). Periostin: a novel component of subepithelial fibrosis of bronchial asthma downstream of IL-4 and IL-13 signals. J. Allergy Clin. Immunol..

[150] Ocklind G, Talts J, Fässler R, Mattsson A, Ekblom P (1993). Expression of tenascin in developing and adult mouse lymphoid organs. J. Histochem. Cytochem..

[151] Saga Y, Tsukamoto T, Jing N, Kusakabe M, Sakakura T (1991). Murine tenascin: cDNA cloning, structure and temporal expression of isoforms. Gene.

[152] Flück M, Chiquet M, Schmutz S, Mayet-Sornay M-H, Desplanches D (2003). Reloading of atrophied rat soleus muscle induces tenascin-C expression around damaged muscle fibers. Am. J. Physiol. Regul. Integr. Comp. Physiol..

[153] Gullberg D (1997). Tenascin-C expression correlates with macrophage invasion in Duchenne muscular dystrophy and in myositis. Neuromuscul. Disord..

[154] Settles DL, Cihak RA, Erickson HP (1996). Tenascin-C expression in dystrophin-related muscular dystrophy. Muscle Nerve.

[155] Zhou S (2020). Myofiber necroptosis promotes muscle stem cell proliferation via releasing Tenascin-C during regeneration. Cell Res..

[156] Wang YX (2019). EGFR-aurka signaling rescues polarity and regeneration defects in dystrophin-deficient muscle stem cells by increasing asymmetric divisions. Cell Stem Cell.

[157] Feige P, Tsai EC, Rudnicki MA (2021). Analysis of human satellite cell dynamics on cultured adult skeletal muscle myofibers. Skelet. Muscle.

[158] Proietti, D. et al. Activation of skeletal muscle-resident glial cells upon nerve injury. *JCI Insight* **6**, e143469. 10.1172/jci.insight.143469 (2021). 10.1172/jci.insight.143469 PMC8119188 33661767

[159] Irintchev A, Salvini TF, Faissner A, Wernig A (1993). Differential expression of tenascin after denervation, damage or paralysis of mouse soleus muscle. J. Neurocytol..

[160] Cifuentes-Diaz C (2002). Abnormal reinnervation of skeletal muscle in a tenascin-C-deficient mouse. J. Neurosci. Res..

[161] Cifuentes-Diaz C (1998). The peripheral nerve and the neuromuscular junction are affected in the tenascin-C-deficient mouse. Cell. Mol. Biol..

[162] Bencze M (2017). Necroptosis, a programmed form of necrosis, participates in muscle degeneration in Duchenne muscular dystrophy. Neuromuscul. Disord..

[163] Morgan JE (2018). Necroptosis mediates myofibre death in dystrophin-deficient mice. Nat. Commun..

[164] Wight TN, Raugi GJ, Mumby SM, Bornstein P (1985). Light microscopic immunolocation of thrombospondin in human tissues. J. Histochem. Cytochem..

[165] De Luna N (2010). Role of thrombospondin 1 in macrophage inflammation in dysferlin myopathy. J. Neuropathol. Exp. Neurol..

[166] Urao N, Mirza RE, Heydemann A, Garcia J, Koh TJ (2016). Thrombospondin-1 levels correlate with macrophage activity and disease progression in dysferlin deficient mice. Neuromuscul. Disord..

[167] Lopez-Dee, Z., Pidcock, K. & Gutierrez, L. S. Thrombospondin-1: multiple paths to inflammation. *Mediat. Inflamm*. **2011**, 296069, 10 (2011). 10.1155/2011/296069 PMC3134184 21765615

[168] Fernández-Simón E (2020). Proteasome inhibitors reduce thrombospondin-1 release in human dysferlin-deficient myotubes. BMC Musculoskelet. Disord..

[169] Vanhoutte D (2016). Thrombospondin expression in myofibers stabilizes muscle membranes. eLife.

[170] Chen Y-W, Zhao P, Borup R, Hoffman EP (2000). Expression profiling in the muscular dystrophies: identification of novel aspects of molecular pathophysiology. J. Cell Biol..

[171] Putnam EA, Zhang H, Ramirez F, Milewicz DM (1995). Fibrillin-2 (FBN2) mutations result in the Marfan-like disorder, congenital contractural arachnodactyly. Nat. Genet..

[172] Sengle G (2015). Abnormal activation of BMP signaling causes myopathy in Fbn2 null mice. PLoS Genet..

[173] Handford PA, Downing AK, Reinhardt DP, Sakai LY (2000). Fibrillin: from domain structure to supramolecular assembly. Matrix Biol..

[174] Hubmacher D, Tiedemann K, Reinhardt DP (2006). Fibrillins: from biogenesis of microfibrils to signaling functions. Curr. Top. Dev. Biol..

[175] Kielty CM (2002). Fibrillin: from microfibril assembly to biomechanical function. Philos. Trans. R. Soc. Lond. B. Biol. Sci..

[176] Carlson ME, Hsu M, Conboy IM (2008). Imbalance between pSmad3 and Notch induces CDK inhibitors in old muscle stem cells. Nature.

[177] Carlson ME (2009). Relative roles of TGF-beta1 and Wnt in the systemic regulation and aging of satellite cell responses. Aging Cell.

[178] Ramirez F, Caescu C, Wondimu E, Galatioto J (2018). Marfan syndrome; a connective tissue disease at the crossroads of mechanotransduction, TGFβ signaling and cell stemness. Matrix Biol..

[179] Dietz HC (1991). Marfan syndrome caused by a recurrent de novo missense mutation in the fibrillin gene. Nature.

[180] Neptune ER (2003). Dysregulation of TGF-beta activation contributes to pathogenesis in Marfan syndrome. Nat. Genet..

[181] Cohn RD (2007). Angiotensin II type 1 receptor blockade attenuates TGF-beta-induced failure of muscle regeneration in multiple myopathic states. Nat. Med..

[182] Gosselin, L. E. Skeletal muscle collagen: age, injury and disease. in *Sarcopenia—Age-Related Muscle Wasting and Weakness: Mechanisms and Treatments* (ed. Lynch, G. S.) 159–172 (Springer Netherlands, 2011). 10.1007/978-90-481-9713-2_8.

[183] Blau HM, Cosgrove BD, Ho ATV (2015). The central role of muscle stem cells in regenerative failure with aging. Nat. Med..

[184] Brack AS, Muñoz-Cánoves P (2016). The ins and outs of muscle stem cell aging. Skelet. Muscle.

[185] Brack AS (2007). Increased Wnt signaling during aging alters muscle stem cell fate and increases fibrosis. Science.

[186] Kimmel, J. C., Hwang, A. B., Scaramozza, A., Marshall, W. F. & Brack, A. S. Aging induces aberrant state transition kinetics in murine muscle stem cells. *Development* **147**, dev183855 (2020). 10.1242/dev.183855 PMC7225128 32198156

[187] Sousa-Victor P (2014). Geriatric muscle stem cells switch reversible quiescence into senescence. Nature.

[188] Muñoz-Cánoves P, Neves J, Sousa-Victor P (2020). Understanding muscle regenerative decline with aging: new approaches to bring back youthfulness to aged stem cells. FEBS J..

[189] Sousa-Victor P, García-Prat L, Serrano AL, Perdiguero E, Muñoz-Cánoves P (2015). Muscle stem cell aging: regulation and rejuvenation. Trends Endocrinol. Metab..

[190] Sousa-Victor P, Neves J, Muñoz-Cánoves P (2020). Muscle stem cell aging: identifying ways to induce tissue rejuvenation. Mech. Ageing Dev..

[191] Sacco, A., Belloni, L. & Latella, L. From development to aging: the path to cellular senescence. *Antioxid. Redox Signal*. 10.1089/ars.2020.8071 (2020). 10.1089/ars.2020.8071 PMC7821433 32228062

[192] García-Prat L (2016). Autophagy maintains stemness by preventing senescence. Nature.

[193] Mann CJ (2011). Aberrant repair and fibrosis development in skeletal muscle. Skelet. Muscle.

[194] Stearns-Reider KM (2017). Aging of the skeletal muscle extracellular matrix drives a stem cell fibrogenic conversion. Aging Cell.

[195] Conboy IM (2005). Rejuvenation of aged progenitor cells by exposure to a young systemic environment. Nature.

[196] Grounds MD (2014). Therapies for sarcopenia and regeneration of old skeletal muscles: more a case of old tissue architecture than old stem cells. Bioarchitecture.

[197] Zhu M (2019). In vivo engineered extracellular matrix scaffolds with instructive niches for oriented tissue regeneration. Nat. Commun..

[198] Rao N (2017). Engineering an injectable muscle-specific microenvironment for improved cell delivery using a nanofibrous extracellular matrix hydrogel. ACS Nano.

[199] Wolf MT, Daly KA, Reing JE, Badylak SF (2012). Biologic scaffold composed of skeletal muscle extracellular matrix. Biomaterials.

[200] Urciuolo A (2018). Decellularised skeletal muscles allow functional muscle regeneration by promoting host cell migration. Sci. Rep..

[201] Chappell L, Russell AJC, Voet T (2018). Single-cell (multi)omics technologies. Annu. Rev. Genomics Hum. Genet..

[202] Wang, X. et al. Three-dimensional intact-tissue sequencing of single-cell transcriptional states. *Science* **361**, eaat56911 (2018). 10.1126/science.aat5691 PMC6339868 29930089

[203] Del Carmen Ortuño-Costela M, García-López M, Cerrada V, Gallardo ME (2019). iPSCs: a powerful tool for skeletal muscle tissue engineering. J. Cell. Mol. Med..

[204] Belleh S (2000). Two novel fibrillin-2 mutations in congenital contractural arachnodactyly. Am. J. Med. Genet..

[205] Lee B (1991). Linkage of Marfan syndrome and a phenotypically related disorder to two different fibrillin genes. Nature.

[206] Tsipouras P (1992). Genetic linkage of the Marfan syndrome, ectopia lentis, and congenital contractural arachnodactyly to the fibrillin genes on chromosomes 15 and 5. The International Marfan Syndrome Collaborative Study. N. Engl. J. Med..

[207] Brinckmann J (2010). Enhanced fibrillin-2 expression is a general feature of wound healing and sclerosis: potential alteration of cell attachment and storage of TGF-beta. Lab. Investig..

[208] Gilpin SE (2017). Fibrillin-2 and tenascin-C bridge the age gap in lung epithelial regeneration. Biomaterials.

[209] Akbareian SE (2013). Enteric neural crest-derived cells promote their migration by modifying their microenvironment through tenascin-C production. Dev. Biol..

[210] Midwood KS, Chiquet M, Tucker RP, Orend G (2016). Tenascin-C at a glance. J. Cell Sci..

[211] Orend G, Huang W, Olayioye MA, Hynes NE, Chiquet-Ehrismann R (2003). Tenascin-C blocks cell-cycle progression of anchorage-dependent fibroblasts on fibronectin through inhibition of syndecan-4. Oncogene.

[212] Saupe F (2013). Tenascin-C downregulates wnt inhibitor dickkopf-1, promoting tumorigenesis in a neuroendocrine tumor model. Cell Rep..

[213] Tucker RP (2001). Abnormal neural crest cell migration after the in vivo knockdown of tenascin-C expression with morpholino antisense oligonucleotides. Dev. Dyn..

[214] Frazier EP (2011). Age-dependent regulation of skeletal muscle mitochondria by the thrombospondin-1 receptor CD47. Matrix Biol..

[215] Audet GN, Fulks D, Stricker JC, Olfert IM (2013). Chronic delivery of a thrombospondin-1 mimetic decreases skeletal muscle capillarity in mice. PloS ONE.

[216] Inoue M (2013). Thrombospondin 1 mediates high-fat diet-induced muscle fibrosis and insulin resistance in male mice. Endocrinology.

[217] Matsugi K, Hosooka T, Nomura K, Ogawa W (2016). Thrombospondin 1 suppresses insulin signaling in C2C12 myotubes. Kobe J. Med. Sci..

[218] Krady MM (2008). Thrombospondin-2 modulates extracellular matrix remodeling during physiological angiogenesis. Am. J. Pathol..

[219] Stenina-Adognravi O, Plow EF (2019). Thrombospondin-4 in tissue remodeling. Matrix Biol..

[220] Frolova EG (2014). Control of organization and function of muscle and tendon by thrombospondin-4. Matrix Biol..

[221] Stupka N (2013). Versican processing by a disintegrin-like and metalloproteinase domain with thrombospondin-1 repeats proteinases-5 and -15 facilitates myoblast fusion. J. Biol. Chem..

[222] Velleman SG, Sporer KRB, Ernst CW, Reed KM, Strasburg GM (2012). Versican, matrix Gla protein, and death-associated protein expression affect muscle satellite cell proliferation and differentiation. Poult. Sci..

[223] Goetsch SC, Hawke TJ, Gallardo TD, Richardson JA, Garry DJ (2003). Transcriptional profiling and regulation of the extracellular matrix during muscle regeneration. Physiol. Genomics.

[224] Kasama T (2005). Neutrophil-derived cytokines: potential therapeutic targets in inflammation. Curr. Drug Targets Inflamm. Allergy.

[225] Butterfield TA, Best TM, Merrick MA (2006). The dual roles of neutrophils and macrophages in inflammation: a critical balance between tissue damage and repair. J. Athl. Train..

[226] Dumont N, Bouchard P, Frenette J (2008). Neutrophil-induced skeletal muscle damage: a calculated and controlled response following hindlimb unloading and reloading. Am. J. Physiol. Regul. Integr. Comp. Physiol..

[227] Scapini P (2000). The neutrophil as a cellular source of chemokines. Immunol. Rev..

[228] Scapini P (2001). Neutrophils produce biologically active macrophage inflammatory protein-3alpha (MIP-3alpha)/CCL20 and MIP-3beta/CCL19. Eur. J. Immunol..

[229] Juban G, Chazaud B (2017). Metabolic regulation of macrophages during tissue repair: insights from skeletal muscle regeneration. FEBS Lett..

[230] Tidball JG, Villalta SA (2010). Regulatory interactions between muscle and the immune system during muscle regeneration. Am. J. Physiol. Regul. Integr. Comp. Physiol..

[231] Burzyn D (2013). A special population of regulatory T cells potentiates muscle repair. Cell.

[232] Arnold L (2007). Inflammatory monocytes recruited after skeletal muscle injury switch into antiinflammatory macrophages to support myogenesis. J. Exp. Med..

[233] Chazaud B (2020). Inflammation and skeletal muscle regeneration: leave it to the macrophages!. Trends Immunol..

[234] Saclier M (2013). Differentially activated macrophages orchestrate myogenic precursor cell fate during human skeletal muscle regeneration. Stem Cells.

[235] Rudolf A (2016). β-Catenin activation in muscle progenitor cells regulates tissue repair. Cell Rep..

